# Interspecific analysis of diurnal gene regulation in panicoid grasses identifies known and novel regulatory motifs

**DOI:** 10.1186/s12864-020-06824-3

**Published:** 2020-06-25

**Authors:** Xianjun Lai, Claire Bendix, Lang Yan, Yang Zhang, James C. Schnable, Frank G. Harmon

**Affiliations:** 1 Center for Plant Science Innovation & Department of Agronomy and Horticulture, University of Nebraska-Lincoln, Lincoln, 68588 USA; 2 College of Agricultural Sciences, Xichang University, Liangshan, Xichang, 615000 China; 3 Department of Plant & Microbial Biology, University of California Berkeley, Berkeley, CA 94720 USA; 4 Plant Gene Expression Center, USDA-ARS, Albany, CA 94710 USA

**Keywords:** Circadian clock, Diurnal rhythms, Evening element, Poaceae grasses, Co-expression cluster, Regulatory motifs, orthologous genes, syntenic genes

## Abstract

**Background:**

The circadian clock drives endogenous 24-h rhythms that allow organisms to adapt and prepare for predictable and repeated changes in their environment throughout the day-night (diurnal) cycle. Many components of the circadian clock in *Arabidopsis thaliana* have been functionally characterized, but comparatively little is known about circadian clocks in grass species including major crops like maize and sorghum.

**Results:**

Comparative research based on protein homology and diurnal gene expression patterns suggests the function of some predicted clock components in grasses is conserved with their *Arabidopsis* counterparts, while others have diverged in function. Our analysis of diurnal gene expression in three panicoid grasses sorghum, maize, and foxtail millet revealed conserved and divergent evolution of expression for core circadian clock genes and for the overall transcriptome. We find that several classes of core circadian clock genes in these grasses differ in copy number compared to *Arabidopsis*, but mostly exhibit conservation of both protein sequence and diurnal expression pattern with the notable exception of maize paralogous genes. We predict conserved *cis*-regulatory motifs shared between maize, sorghum, and foxtail millet through identification of diurnal co-expression clusters for a subset of 27,196 orthologous syntenic genes. In this analysis, a Cochran–Mantel–Haenszel based method to control for background variation identified significant enrichment for both expected and novel 6–8 nucleotide motifs in the promoter regions of genes with shared diurnal regulation predicted to function in common physiological activities.

**Conclusions:**

This study illustrates the divergence and conservation of circadian clocks and diurnal regulatory networks across syntenic orthologous genes in panacoid grass species. Further, conserved local regulatory sequences contribute to the architecture of these diurnal regulatory networks that produce conserved patterns of diurnal gene expression.

## Background

Genes exhibiting rhythmic patterns of expression under day-night, or diurnal, conditions are widespread in plants as in other domains of life. The expression patterns of these cycling genes are shaped by both internal signals from the circadian clock and external environmental cues [1]. A fundamental role of biological rhythms is to ensure that different physiological processes occur at the most favorable time of day thereby optimizing growth throughout the day-night cycle [2].

A primary mechanism driving biological rhythms is transcriptional control [3]. Transcriptome-wide analysis in diverse plant species reveals diurnal rhythmic expression for 25–60% of transcripts in leaves of maize, rice, popular, and Brachypodium [4–6], 30% of transcripts in conifer needles [7], and up to 89% of transcripts in whole *Arabidopsis thaliana* seedlings [1]. The circadian clock and direct responses to environmental cues together provide transcriptional signals to a highly interconnected regulatory network that shapes the temporal behavior of key plant signaling and metabolic pathways. Previous work identified conserved *cis*-elements upstream of diurnal rhythmic genes in *Arabidopsis*, poplar, and rice [1], indicating conserved regulatory *cis*-elements are critical to shaping diurnal rhythms. The regulatory logic shaping rhythmic gene expression and how diurnal transcriptional networks impose timing on biological processes are not fully understood.

The circadian clock system allows organisms to anticipate daily changes in light and temperature, as well as seasonal transitions associated with changes in daylength [8–10]. The circadian clock is an endogenous and self-sustaining mechanism generating approximately 24-h rhythms in biological processes. The rhythms generated by circadian clocks allow organisms to anticipate recurring environmental changes. For example, movement of sunflower heads to face east before the sun rises [11]. Coordination of internal physiological activities with external environmental conditions mediated by the circadian clock maximizes biomass and growth vigor [9, 12–14].

The circadian clock regulates metabolic pathways involved in plant growth, development, and biotic stress tolerance [15, 16]. Natural variants of the GIGANTEA gene in *Brassica rapa* alter circadian clock period and contribute to differences in cold and salt stress tolerance [17]. Cultivated tomato accessions have slower circadian clocks compared to wild tomato accessions [18]. This change in circadian clock activity is associated with increased plant height, earlier flowering, and reduced chlorophyll content, which adapted cultivated tomato to the longer summer days of higher latitudes. Early flowering caused by disrupted circadian clock activity allows cultivation of certain diploid wheat and barley cultivars under the short growing seasons at high latitudes [19–21]. Circadian clock activity in *Arabidopsis* and maize hybrids plays an essential part in the origin of hybrid vigor [13, 22]. Metabolic vigor in *Arabidopsis* hybrids and allopolyploids partly results from temporal shifts in circadian clock regulation of key metabolic genes [13]. Similarly, higher levels of carbon fixation and starch accumulation in maize hybrids are associated with an altered phase of circadian gene expression [22].

Core circadian clock genes initially discovered in *Arabidopsis* occur throughout the green plant lineage. The complement of predicted circadian clock genes in *B. rapa* is comparable to *Arabidopsis*, although gene copy number varies between the two species, primarily as a result of local gene duplication in *B. rapa* [17, 23]. Homologous circadian clock genes have been identified in other eudicots including tomato [24], wild tobacco [25], grapes [26] and multiple legumes [27, 28]. Monocots such as maize [29], wheat [21], barley [20, 30], and rice [5, 31, 32] also have homologs of *Arabidopsis* circadian clock genes.

Domesticated and wild grass species in the Poaceae family have adapted to diverse environments, but species within this family exhibit significant syntenic conservation at the level of both genetic maps and genomic organization [33–35]. While a minority of annotated maize genes are conserved at syntenic locations relative to other grass species of Poaceae, these syntenic conserved genes account for the vast majority of genes with known mutant phenotypes [36]. Several studies have identified conservation of both diurnal gene expression patterns and *cis*-regulatory elements implicated in the regulation of these expression patterns across related species [13, 22, 37, 38].

We examined the conservation and divergence of diurnal gene regulation among syntenic orthologs from the panacoid grasses sorghum (*Sorghum bicolor*), maize (*Zea mays*), and foxtail millet (*Setaria italica*) to identify *cis*-regulatory elements with potentially conserved and divergent functions in shaping diurnal gene expression. Shared diurnal regulation patterns among syntenic orthologs in related species indicates the subset of diurnal gene regulatory patterns experiencing the greatest degree of functional constraint. Our expectation was that conserved regulatory *cis*-elements responsible diurnal expression patterns will be found upstream of these co-expressed genes. We first identified all genes experiencing diurnal regulation in each grass species with transcriptome-wide evaluation of diurnal gene expression with RNA-seq of samples taken across a 3-day time course. We next identified gene families for circadian clock components based on homology to known *Arabidopsis* circadian clock components and evaluated the expression behavior of these genes to determine conserved features of circadian clock regulation between these three grasses. Finally, we identified highly credible conserved upstream *cis*-motifs shared by maize, sorghum, and foxtail millet employing a cluster-based method that takes advantage of conservation information from multiple species. This analysis discovered several well-known and novel DNA sequence motifs that were enriched in upstream regions of genes involved in the same metabolic pathways. We conclude that conserved local regulatory sequences contribute to the architecture of these diurnal regulatory networks that produce conserved patterns of diurnal gene expression.

## Results

### Transcriptome-wide diurnal expression in sorghum, maize and foxtail millet

To identify the subset of genes experiencing diurnal patterns of regulation and to compare the characteristics of diurnal regulatory patterns across orthologous genes, diurnal expression was characterized for sorghum, maize, and foxtail millet, three closely related grass species. Fully expanded third leaves from sorghum, maize, and foxtail millet plants at the 3 leaf stage were sampled every 3 hours over the course of 3 days (a total of 72 h) (Figure S1). The resulting RNA-seq-based gene expression profiling datasets are summarized in Table S1. As expected, a large proportion of genes in each of the three species exhibited rhythmic expression. Curve fit analysis to identify rhythmic gene expression patterns showed 52% (16,752 rhythmic/32,446 total), 30% (17,532 rhythmic/59,074 total), and 43% (15,046 rhythmic/34,680 total) of detected transcripts in sorghum, maize and foxtail millet, respectively, had statistically significant diurnal rhythms (Tables S2-S4). For each gene with significant evidence of rhythmic expression, the pattern of expression was described using three variables: period (the time required to complete 1 cycle), phase (the time of peak expression), and amplitude (the difference between peak and trough of expression). The majority of cycling genes exhibited a period of 24 h (75% sorghum, 86% maize, 77% foxtail millet; Tables S2, S3, S4), as expected for diurnal regulation and consistent with the light-dark environmental conditions experienced by the plants. Within each 24-h period, the phase distribution was continuous, meaning that at every time of day peak expression occurred for many different genes (Tables S2, S3, S4). In all three species, the most common phases were between afternoon, corresponding to circadian time (CT) 9 (9 h after dawn), and early morning (CT18) (Figure S2). The collection of genes expressed in this 9 h time interval represented 74.5% (sorghum), 71.2% (maize), and 75.5% (foxtail millet) of all cycling genes. The median amplitude for all rhythmic genes was 5.5 (sorghum), 4.9 (maize) and 5.5 (foxtail millet), but genes with amplitudes < 5 made up 50% (sorghum), 54.5% (maize), and 49.9% (foxtail millet) of the values (Figure S3). Overall, the proportion of genes exhibiting diurnal regulation and the distribution of phases for maize, sorghum, and foxtail millet observed here are similar to the nature and extent of diurnal regulation described for other flowering plant species [4–6, 14, 29].

### Conserved expression for predicted core circadian clock genes

**Fig. 1:**
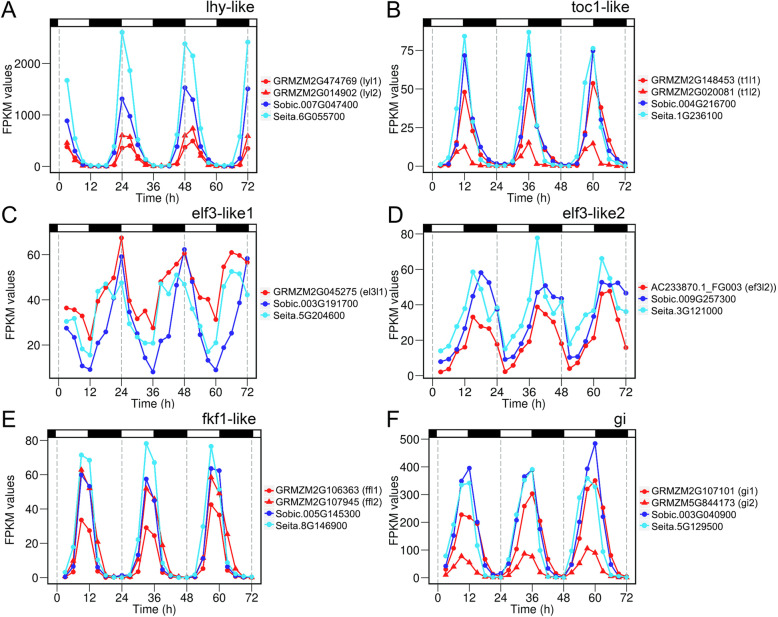
Diurnal expression patterns of orthologous central circadian clock genes from maize, sorghum and foxtail millet over a 72-h. Expression patterns of (**a**) *lhy-like* (*lyl*), (**b**) *toc1-like* (*t1l*), (**c**, **d**) *elf3-like* (*el3l*), (**e**) *fkf1-like* (*ffl*) and (**f**) *gigantea* (*gi*) genes. *lyl1* FPKM values are for gene model *GRMZM2G474769*, but the complete *lyl1* gene encompasses three annotated genes (Table S6). The two maize paralogous *el3l1* and *el3l2* are presented separately together the sorghum and foxtail millet orthologs. Sorghum genes shown in by blue, foxtail millet genes in light blue, and maize genes in red. Maize paralogs are indicated by circle and triangle symbols. White and black bars correspond to times of light and dark, respectively

To test how consistently the core circadian clock behaves across these three grasses, we focused on a set of genes encoding orthologs of *Arabidopsis* circadian clock components. Putative orthologs were identified in maize using the amino acid sequences of the *Arabidopsis* proteins (Tables S5, S6) [9, 39, 40]. Shared synteny was used to establish orthology to map homologous relationships over to the sorghum and foxtail millet genomes and to identify pairs of maize genes which are homeologous duplicates resulting from the maize/*Tripsacum* whole genome duplication (WGD) event (Table S7) [41]. We found these circadian clock components have similar diurnal expression patterns in maize, sorghum, and foxtail millet, but several orthologs show advanced or delayed peak expression in maize (Figs. 1, S7; Table 1). Analysis of five key circadian clock gene families is described below.

**Table 1:** Diurnal expression characteristics of syntenic circadian clock and circadian clock-associated genes

		CT Phase^a^, hours					Amplitude^a^, FPKM		
Name	At^b^	Sb	Zm1	Zm2	Si	Sb	Zm1	Zm2	Si
LYL1/LHYL2	23	0	1.5	0	0	218.1	33.8	31.9	385.7
RE2L	20	0	21	-^c^	22.5	52.6	18.8	–	65.0
RE6L1	nd^d^	21	21	–	21	8.4	1.3	–	4.8
RE6L2/RE6L3	nd	nr^e^	nr	nr	18	nr	nr	nr	3.7
RE6L4/RE6L5	nd	22.5	21	22.5	21	24.4	3.0	18.1	44.4
RE7L1/RE7L2	nd	19.5	18	14.7	19.5	1.9	2.3	0.8	1.8
RE7L3/RE7L4	nd	15	16.5	18	13.5	10.8	23.3	18.9	37.5
T1L1/T1L2	13	10.5	12	9	9	9.5	5.9	1.1	5.6
P59L1	8	–	7.5	–	–	–	2.2	–	–
P59L2	8	8.3	–	9	7.5	14.3	–	3.4	10.2
P73L	7	6	–	4.5	4.5	74.3	–	45.3	66.9
P95L1/P95L2	5	6	6	7.5	6	21.0	16.0	2.5	45.2
GI2/1	8	9	7.5	9	7.5	120.4	13.0	103.1	135.2
EF3L1	14	21	20.3	–	19.5	10.8	6.8	–	11.0
EF3L2	16	16.5	15	–	13.5	27.9	12.1	–	16.2
EF4R1/2	11	9	7.5	nr	7.5	14.5	9.4	nr	35.9
EF4R3	11	nr	nr	–	nr	nr	nr	–	nr
EF4R4	11	10.6	13.5	–	9.0	0.6	1.5	–	1.5
LXL	11	12	–	10.5	10.5	4.7	–	5.6	12.9
ZLL1/ZLL2	nr	16.5	nr	10.5	16.5	7.8	nr	1.7	4.8
ZLL3/ZLL4	nr	0	0	nr	15	10.3	4.5	nr	3.1
FFL1/FFL2	11	7.5	8.3	9	7.5	3.8	2.3	7.2	8.3

^a^Values are from JTK_Cycle analysis in Tables S2-S4 for sorghum (Sb), maize subgenome 1 (Zm1), maize subgenome 2 (Zm2), and foxtail millet (Si) genes

^b^Phase values for orthologous *Arabidopsis* (At) genes plants under light-dark photocycles and hot-cold temperature cycles (LDHC) from reference [37]

^c^No syntenic gene

^d^Not determined

^e^Expression not rhythmic

#### LHY/CCA1 and RVE genes

*Arabidopsis* CCA1 and LHY and their mutual grass orthologs form a monophyletic clade within the larger REVEILLE (RVE) protein family (Figure S4A). LHY/CCA1 are Myb-like transcription factors that are central components of all plant circadian clock models [39, 40]. Previous analysis of the CCA1 and LHY phylogeny in eudicots identified that the duplication that produced the *CCA1* and *LHY* genes occurred in the Brassicaceae lineage, after the eudicot/monocot split [23]. Hence, any grass gene apparently orthologous to *CCA1* shares an equal orthologous relationship with *LHY* and vice versa. Maize harbors two *LHY-like* (*lyl*) genes caused by the maize/Tripsacum WGD event, while sorghum and foxtail millet each have a single *lyl* gene that each are co-orthologous to the maize gene pair according to a phylogenetic tree of LYL protein family (Figure S5). The sorghum (*Sobic.007G047400*), maize (*GRMZM2G474769*; *GRMZM2G014902*) and foxtail millet (*Seita.6G055700*) *lyl* genes displayed clear rhythmic expression patterns (Fig. 1a). The sorghum and foxtail millet genes exhibited higher peak expression and greater amplitude than their two maize orthologs, but the two maize gene copies were expressed at equivalent levels and amplitude relative to each other (Table 1). Also, peak expression of the sorghum and foxtail millet *lyl* genes occurred coincident with dawn, while the maize *lyl* peak occurred 3 h later. The morning-phased expression of these *lyl* genes is similar to *Arabidopsis LHY* and *CCA1* (Table 1).

At the protein level, all of the maize LHY-like and RVE-like proteins share the same amino acid domains as their *Arabidopsis* counterparts, including the signature Myb-type DNA-binding domain (Tables S5, S6). The five other subclades of syntenic orthologous genes in the RVE gene family, *RVE2-like* (*re2l*), *RVE6-like* (*re6l*), and *RVE7-like* (*re7l*), showed strong rhythmic expression across the three species (Figure S7A-S7; Table 1). Genes of the *re2l* family in all three species, including the single maize gene produced by genome fractionation (*GRMZM2G145041*), also exhibited a peak in expression at dawn (Figure S7A; Table 1). This pattern is also consistent with *re6l1* in maize (*GRMZM2G135052*) and orthologs in sorghum (*Sobic.010G004300*) and foxtail millet (*Seita.4G004600*) (Figure S7B; Table 1). On the other hand, the orthologous gene group with maize *re6l2* (*GRMZM2G170148*) and *re6l3* (*GRMZM2G057408*) and their sorghum ortholog (*Sobic.010G223700*) is not rhythmically expressed, while the foxtail millet gene (*Seita.4G266800*) has rhythmic expression with peak expression at dawn (Figure S7C; Table 1). Maize *re6l4* (*GRMZM5G833032*) and *re6l5* (*GRMZM2G118693*) together with its sorghum (*Sobic.004G281800*) and foxtail millet (*Seita.1G272700*) orthologs show rhythmic expression with a dawn phase, but maize *re6l4* expression levels and amplitude are low (Figure S7D; Table 1). The orthologous gene groups of *re7l* genes were rhythmically expressed, but the first group, representing maize homeologous genes *re7l1* (*GRMZM2G029850*) and *re7l2* (*GRMZM2G170322*), exhibited dramatically lower gene expression than orthologs in sorghum (*Sobic.004G279300*) and foxtail millet (*Seita.1G275400*) (Figure S7E; Table 1). In the second orthologous group of *re7l* genes, the phase of the foxtail millet gene expression (*Seita.7G212900*) was shifted earlier in the night, with a broader peak, while both maize *re7l3* (*GRMZM2G421256*) and *re7l4* (*GRMZM2G181030*) showed a sharp peak in expression coincident with dawn, and the sorghum peak (*Sobic.006G192100*) was intermediate between the two (Figure S7F; Table 1).

#### PSEUDO-RESPONSE REGULATOR (PRR) genes: TOC1, PRR3/7/37/73, PRR9/5,95

The *Arabidopsis PSEUDO-RESPONSE REGULATOR* (*PRR*) genes *PRR9*, *PRR7*, *PRR5*, and *TIMING OF CAB EXPRESSION 1* (*TOC1*) encode core circadian clock components that repress transcription of *CCA1*/*LHY* throughout the day [42]. The proteins in the PRR family fall into three main clades named TOC1, PRR3/7/37/73, and PRR9/5/95 (Figure S4B). PRR-like proteins in all three species contain the same amino acid motifs as the *Arabidopsis* PRRs (Tables S5, S6). Interestingly, sorghum and foxtail millet have single copies of *TOC1-like* (*t1l*), PRR73-like (*p73l*), *PRR95-like* (*p95l*) and *PRR59-like* (*p59l*) genes while maize also has a single *p73l* gene but two each of the *t1l*, *p95l*, and *p95l* genes (Table S6). All of these maize genes arose from recent duplications (Table S7). Diurnal gene expression for all three species exhibited peak expression at midday for *p73l* and *p95l* genes (Figure S7G, I), at late-afternoon for *p59l* genes (Figure S7H), and at dusk for *t1l* genes (Fig. 1b), which is a pattern similar to their Arabidopsis orthologs (Table 1).

#### Evening complex genes: ELF3, ELF4, and LUX

The evening complex is a trimeric protein complex that contains EARLY FLOWERING 3 (ELF3), EARLY FLOWERING 4 (ELF4), and the Myb-like transcription factor LUX ARRHYTHMO (LUX). This protein complex represses expression of day-phased genes [43]. The maize genome has two *ELF3-like* (*elfl*) genes (*GRMZM2G045275*, *AC233870.1_FG003*) encoding complete EF3L proteins (Table S6), although the domains of ELF3 proteins do not represent conserved domains of known function (Table S5). These *elfl* genes appear to have arisen from gene duplication in the common monocot WGD event, as each of the maize *elfl* genes is on unfractionated maize subgenome 1 and neither has a direct paralog on maize subgenome 2 (Tables S6, S7). The *ef3l* genes in all three grasses had consistently high amplitude rhythms, although the daily timing of peaks was different amongst them (Fig. 1c, d; Table 1). Maize *ef3l1* and its orthologs from sorghum (*Sobic.003G191700*) and foxtail millet (*Seita.5G204600*) reached peak expression around dawn (Fig. 1c). On the other hand, maize *ef3l2* and its orthologs from sorghum (*Sobic.009G257300*) and foxtail millet (*Seita.3G121000*) peaked early in the night, which is timing similar to *Arabidopsis* ELF3 (Fig. 1d; Table 1).

Single *LUX-like* (*lxl*) genes occur in sorghum, maize and foxtail millet (Tables S6, S7). The grass LXL proteins have Myb-like DNA binding domains homologous to *Arabidopsis* LUX, but are substantially shorter than *Arabidopsis* LUX by 13, 33 and 22% for sorghum, maize, and foxtail millet, respectively. Rhythmic expression of *lxl* in maize (*GRMZM2G067702*) and foxtail millet (*Seita.5G468100*) was at higher levels than sorghum *lxl* (*Sobic.003G443600*) (Figure S7J), but all the genes had maximal expression at dusk like *Arabidopsis LUX* (Table 1).

*Arabidopsis ELF4* belongs to a family also containing four ELF4-LIKE (EF4L*)* proteins [44]*.* ELF4 and EF4L1 are members of one subclade (ELF4/EF4L1 clade), while EF4L2, EF4L3, and EF4L4 belong to another (EF4L2/3/4) (Figures S4C, S6). The nomenclature ELF4-RELATED (EF4R) is used here for the monocot proteins to distinguish them from dicot ELF4L proteins. We find the ELF4/EF4L1 subclade contains only proteins from dicots (Figure S6A). Several grass EF4R proteins fall within a separate subclade (Figures S4C; S6A), including two sorghum proteins (Sobic.005G194200 and Sobic.002G193000) and their two maize orthologs EF4R3 (GRMZM5G877647*)* and EF4R4 (GRMZM2G025646), each encoded by genes that have lost their paralogs (Table S7). A separate, potentially monocot-specific, clade is basal to the others and contains EF4R1 (GRMZM2G382774) and EF4R2 (GRMZM2G3593222), which are encoded by paralogous genes (Tables S6, S7), together with proteins from sorghum (Sobic.001G340700) and foxtail millet (Seita.2G195800) (Figure S6B).

Of *ef4r* genes, *ef4r1* from maize, sorghum, and foxtail millet were the most highly expressed rhythmic genes and these had peak expression late in the late day (Figure S7K; Table 1). By contrast, the expression level of *ef4r2*, which is the paralog of maize *ef4r1*, was not sufficient to detect rhythms (Figure S7K). Each of the *ef4r3* orthologs from sorghum, maize, and foxtail millet was expressed, but these did not have rhythmic expression (Figure S7L; Table 1). Finally, the *ef4r4* orthologs from all three species had low expression levels characterized by low amplitude rhythms (Figure S7M; Table 1).

#### ZTL, LKP2, and FKF1 genes

The three closely related genes *ZEITLUPE* (*ZTL*)/*ADAGIO 1* (*ADO1*), *LOV KELCH PROTEIN 2* (*LKP2*)/*ADAGIO 2* (*ADO2*), and *FLAVIN-BINDING*, *KELCH REPEAT*, *F-Box 1* (*FKF1*)/*ADAGIO 3* (*ADO3*) encode blue light photoreceptors involved in ubiquitin-26S proteasome-directed protein turnover [45]. ZTL primarily contributes to clock function, while FKF1 and LKP2 are involved in photoperiodic control of flowering time. The *LKP2* gene group is present only in the Brassicaceae lineage [23] and, therefore, was not considered here.

Foxtail millet and sorghum each have two *ZTL-like* (*zll*) genes and maize has four *zll* genes (Table S6). Maize *zll1* (*GRMZM2G115914*) and *zll2* (*GRMZM2G113244*) are paralogs syntenic to individual sorghum (*Sobic.010G243900*) and foxtail millet (*Seita.4G249100*) genes (Table S7). Similarly, maize *zll3* (*GRMZM2G147800*) and *zll4* (*GRMZM2G166147*) are paralogs syntenic to individual sorghum (*Sobic.004G042200*) and foxtail millet (*Seita.1G087300*) genes (Tables S6, S7). The expression behavior of these *zll* genes is complex. Maize *zll1* and *zll4* appear not to be expressed (Figure S7N, O; Table 1). Intriguingly, maize *zll2* has peak expression at dusk, while expression of its sorghum and foxtail millet orthologs occurs 6 h later in the middle of the night. Maize *zll3* and its sorghum ortholog both achieve peak expression at dawn; in contrast, the phase of their foxtail millet ortholog is 15 h later in the middle of the night period similar to the second foxtail millet *zll* gene (Figure S7O).

Sorghum and foxtail millet have single *FKF1-like* (*fflI) FFL* genes (*Sobic.005G145300* and *Seita.8G146900*) and maize has the two paralogous *genes ffl1* (*GRMZM2G106363*) and *ffl2* (*GRMZM2G107945*) (Tables S6, S7). The *ffl* genes in sorghum, maize and foxtail millet all reach peak expression in the mid-afternoon. Amplitude was similar between maize *ffl1* and sorghum *ffl*, but the amplitudes of maize *ffl2* and foxtail millet *ffl* were higher by nearly 2-fold (Fig. 1e; Table 1).

#### GI genes

The *Arabidopsis GIGANTEA* (*GI*) gene encodes a plant-specific protein that interacts with ZTL/FKF1 proteins to control their degradation by the ubiquitin-26S proteasome system [46, 47]. Maize has the paralogous *gi1* (*GRMZM2G107101*) and *gi2* (*GRMZM5G844173*) genes on subgenome 1 and subgenome 1, respectively (Tables S6, S7). By contrast, sorghum (*Sobic.003G040900*) and foxtail millet (*Seita.5G129500*) each have a single *gi* gene. As reported previously [48], maize *gi1* expression level and amplitude was higher than maize *gi2*, but both are expressed late in the day similar to the sorghum and foxtail millet *gi* genes (Fig. 1f; Table 1).

### Identification of interspecies co-expression based on K-means clustering

Orthologous syntenic genes are sets of genes located in genomic regions derived from the same ancestral genomic region and in a collinear gene order across genomes. These genes in sorghum, maize and foxtail millet are expected to have consistent behaviors under the same external environment. Many of the predicted core circadian clock genes described above showed diurnal expression patterns that are expected based on the behavior of their *Arabidopsis* orthologs (Table 1), although several had shifted expression phases or the absence/presence of rhythmicity. To investigate conserved temporal regulation of gene expression transcriptome-wide for sorghum, maize, and foxtail millet, we identified shared overall expression patterns amongst syntenic genes using a K-means clustering method. The expectation was that orthologous syntenic genes derived from the same ancestral genomic regions will preserve key regulatory features and, as a consequence, retain comparable expression behavior under equivalent conditions. Thus, syntenic genes from sorghum, maize, and foxtail millet are expected to be grouped together according to expression pattern at a substantially higher frequency than chance. The existence of such co-expression clusters implies coordinated regulation for genes within the cluster.

For K-means clustering, an orthologous syntenic gene subset consisting of 57,802 total genes from sorghum, maize, and foxtail millet (Table S7) was extracted from a pan-grass syntenic gene set [49] and was used to construct a gene expression matrix based on the 72-h time series RNA-seq datasets. To remove genes with low gene expression reproducibility and to maximize the number of syntenic genes analyzed, K-means clustering analysis considered a subset of 27,196 total orthologous genes (8616 sorghum genes, 8836 foxtail millet genes, and 9744 maize genes) that corresponded to syntenic gene groups having a Pearson correlation higher than 0.7 and a mean signed deviation (MSD) lower than 0.9 between successive days.

**Fig. 2:**
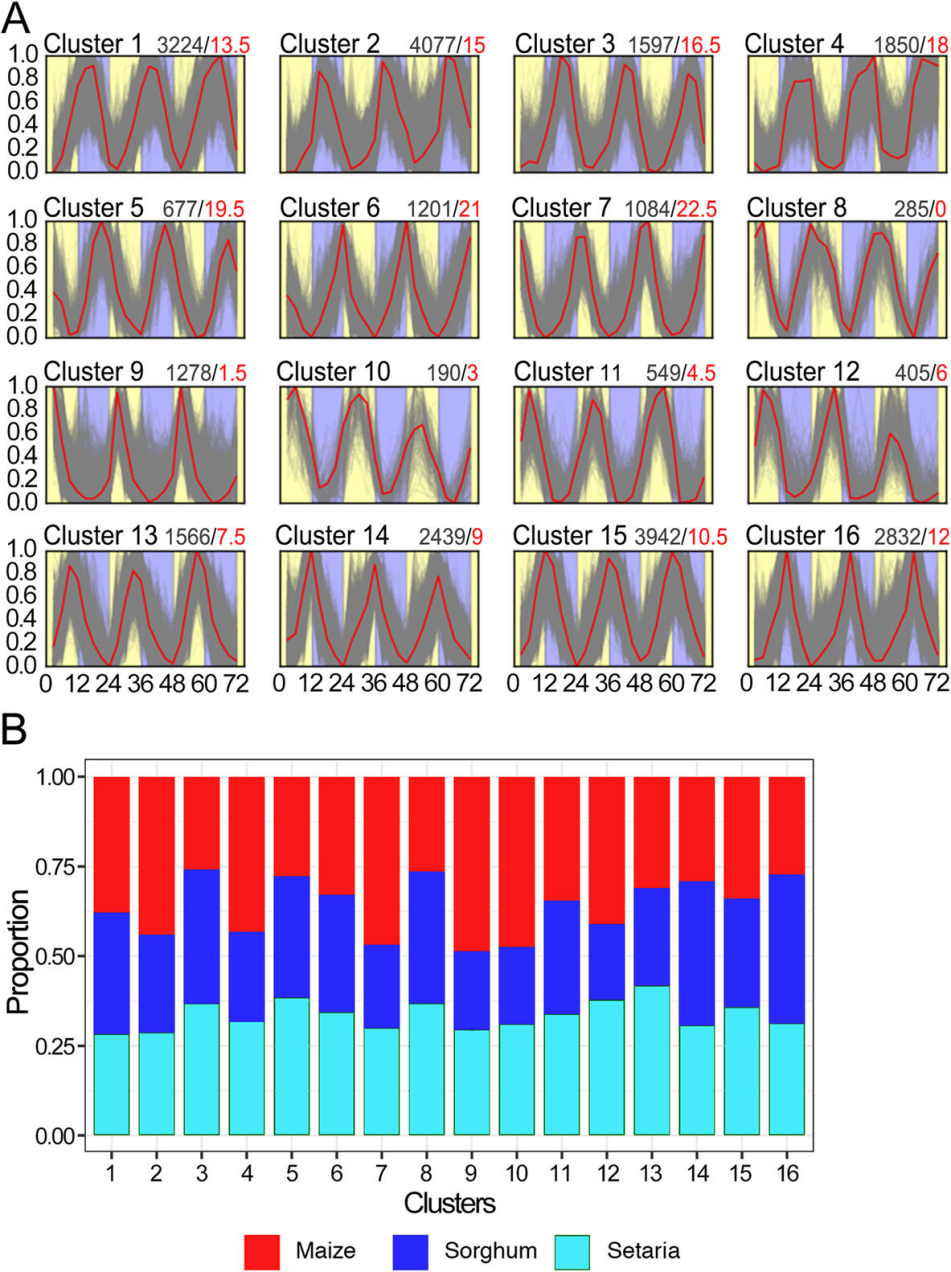
Diurnal co-expression clusters for orthologous syntenic sorghum, maize, and foxtail millet genes. **a** Normalized gene expression values show the diurnal expression pattern in 16 clusters derived from K-means clustering. **b** Proportion of genes from maize (red), sorghum (dark blue), and foxtail millet (light blue) in each of the 16 clusters. In (**a**), x-axis shows the time points and y-axis shows the 0–1 scaled FPKM values for each gene. Yellow and blue areas represent light and dark periods, respectively. Heavy red line is the median expression pattern of genes in that cluster and fine grey lines are individual genes. The total number of genes and median phase (in CT) of each cluster are show at the top of each panel as: total gene number/median phase (in red text)

To identify a reasonable number of K-means clusters representing distinct gene expression patterns, we tested a series of candidate cluster centers from 2 to 24 to discover the number of K-means centers that both clustered the highest number of syntenic sorghum and foxtail millet orthologs and minimized the false discovery rate (FDR) (Figure S9). Based on the expectation that orthologous genes could be grouped in the same cluster by chance, we conducted a permutation test 100 times, each time shuffling the assignment of genes to clusters and calculated the average expectation values from all permutations. In the case of random distribution, the true positive ratio, or the percentage of orthologous genes appearing together in a cluster, will be inversely proportional to the number of clusters leading to a higher FDR with fewer clusters. In our permutation analysis, we found that the true positive ratio fell substantially between 2 and 15 centers but then plateaued when the cluster number exceeded 15 (Figure S9). Taking this result into consideration with the fact the dataset had 8 time points representing a full day-night cycle, we grouped the orthologous genes into 16 clusters in which two distinct expression patterns could be present for the same time point (Table S8). A total of 2278 syntenic gene pairs between sorghum and foxtail millet were enriched in clusters, accounting for 37.2% of all the syntenic gene groups. All clusters were composed of genes with clear diurnal expression patterns (Fig. 2a). Notably, the clusters had distinct median phases of expression, indicating these clusters were potentially groups of co-regulated genes, which may be related to specific biological processes with distinct diurnal rhythms.

Given the high genomic collinearity across these three grasses species [33–35], the number of genes enriched in the same clusters across species were expected to be equivalent. A comparison of gene distributions for each species demonstrated generally uniform gene composition in each cluster (Fig. 2b), except for six clusters with a slightly greater number of maize genes (clusters 2, 4, 7, 9, 10, and 12). This bias is likely explained by the presence of duplicated maize paralogs, since these clusters in total had a significant higher proportion of duplicated maize paralogs than the total of the other clusters (t-test p-value = 2.83e-5). The composition of these clusters is in line with our hypothesis regarding conserved co-regulation of genes and also demonstrates the utility of K-means clustering in identification of co-expressed genes between diverse species.

### Characteristics of orthologous gene expression patterns

**Fig. 3:**
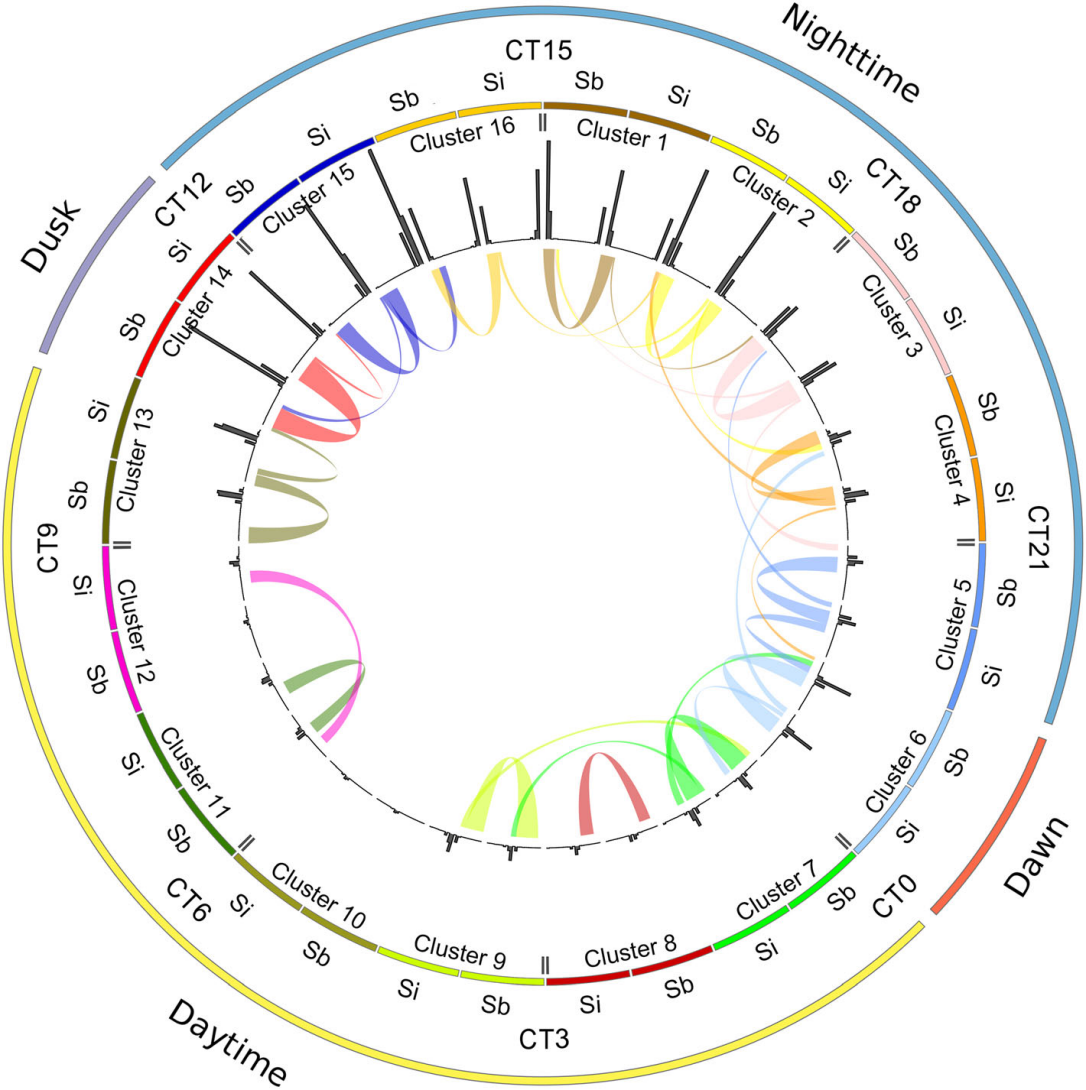
Phase distribution for orthologous genes with diurnal expression from sorghum and foxtail millet. Concentric circles represent different features layered together from the outside to inside. The outer layer represents dawn (red), daytime (yellow), dusk (purple), and nighttime (blue) and the corresponding CT for each part of the diurnal cycle. CT0 is equal to 9:00 AM. The second layer shows the relative temporal position of the median gene expression for sorghum and foxtail millet orthologs in the 16 co-expression clusters, where each color represents one cluster. The third layer shows the 24-h temporal distribution of peak expression for genes in that cluster along a line beginning and ending at CT12 (midnight). The inner most layer shows the cluster position of enriched syntenic genes shared between sorghum and foxtail millet based on the null model. Each colored line represents at least 10 shared genes and line thickness is proportional to the number of shared genes

Orthologous syntenic sorghum, maize, and foxtail millet genes were expected to group in the same clusters, an indication of conserved transcriptional regulatory mechanisms. To determine whether orthologous syntenic genes were enriched in clusters over non-syntenic genes, we investigated the proportion of syntenic genes in the clusters. Since the distribution of phases in clusters spans several hours, the median phase in each cluster was used to represent the center phase of the cluster. The phases of the16 clusters were distributed across the 24-h diurnal period with a 1.5-h interval between two temporally adjacent clusters (Fig. 2a). Pairwise comparisons were made between orthologous genes of two species to detect conserved and divergent gene expression patterns based on phase of gene expression (Figs. 3 and S10, S11). In total, 37.2% of orthologous genes appeared in the same clusters, which indicated the conservation of diurnal expression patterns for these genes. Even in cases when syntenic genes that did not appear in the same cluster, conserved expression patterns were apparent by the clustering of syntenic genes in temporally similar diurnal phases. Orthologous genes grouped in different clusters were more likely to be in a cluster representing a similar phase than expected based on our null model of random distribution (Figs. 3, S10, S11). The phase distribution for expression of clustered genes was also highly conserved (Fig. 2a). Similar to the distribution observed with curve fit analysis (Figure S2), clusters with the greatest numbers of genes were those representing genes with peak expression between afternoon (CT9) and early morning (18 h after dawn), which encompassed in total 73.4% of all genes in clusters, while only 9.9% of genes fell into clusters representing genes expressed from the beginning of the day (CT0) to the afternoon (CT6).

### Diurnal co-regulation of genes related by function

GO enrichment analysis was performed to identify functional enrichment of genes in the 16 co-expression clusters (Table S9). A total of 1200 GO Cellular Process terms were enriched for each species, of which ~ 200 s were significant enriched (Bonferroni adjusted p-value *<* 0.05). Clusters 8–12 represent genes with morning/daytime phases (CT0–6) were enriched for GO terms of “photosynthesis light harvesting”, “heme binding”, “tetrapyrrole binding”, and “generation of precursor metabolites and energy”. This demonstrates coordinated regulation of photosynthesis- and metabolism-related genes to ensure these are expressed during the day when plants are actively engaged in photosynthesis. Genes in cluster 14, with phases near the boundary of day and night (CT9), represented GO terms related to basic cellular functions, including ribosome biosynthesis, protein translation, macromolecule biosynthetic process, and nitrogen metabolism. Clusters 15 and 16, representing genes with evening timing (CT10.5-CT12), were also enriched for genes related to protein synthesis including nitrogen compound metabolism, ribosome synthesis, and RNA synthesis. Nighttime and early morning (CT13.5–19.5) expressed genes in clusters 1–5 were mainly related to a range of metabolic process including nucleic acid, peptide, and organic cyclic compounds, as well as phosphorylation, and oxidoreductase activity, which is likely associated with degradation of dissolved sugars and starch throughout the night [50]. GO terms related to chromatin binding and regulation of RNA biosynthetic processes and gene expression were enriched in clusters 6 and 7 that represent genes expressed just before dawn (CT21–22.5). The co-expression of syntenic genes associated with specific cellular activities demonstrates conserved diurnal partitioning of cellular activities in sorghum, maize, and foxtail millet.

### Leveraging multi-species comparisons for increased power to identify promoter motifs

Given the hypothesis that genes with common expression patterns are controlled by shared regulatory mechanisms, the diurnal co-expression gene clusters identified here offered an opportunity to identify promoter motifs conserved between the sorghum, maize, and foxtail millet genes. All permutations of 6–8 nucleotides were regarded as putative motifs and we searched for motifs shared between genes in each co-expression cluster in likely promotor regions, corresponding to 1000 base pairs upstream from the transcriptional start site. Initially, the Fisher’s exact test was applied to determine if any motif was significant enriched in any cluster for each species. A permutation-based FDR was calculated to control the proportion of false positives. With this approach, only two 6 nucleotide motifs were found significantly enriched in the same clusters across the three species and no motifs of 7 or 8 nucleotides were found (Table S10). One of these two motifs is ‘AATATC’ in cluster 2 and it is the core sequence of the evening element (EE) [8], which is the binding target of *Arabidopsis* LHY and CCA1 [51]. The second enriched motif is ‘GGCCCA’ present in cluster 5, which represents early morning-phased genes, and this sequence matches the *Arabidopsis* UP1ATMSD motif previously discovered associated with protein synthesis-related genes [52, 53]. The limited number of motifs discovered by this Fisher’s test method, indicated it was inefficient at identifying motifs in a single species, as well as motifs shared amongst species.

To improve motif discovery, we performed the Cochran–Mantel–Haenszel (CMH) test. This test combines separate 2 × 2 contingency tables for each species, which controls for variation in the background abundance of distinct sequence nucleotide motifs in the promoters of orthologous genes in different species. Analysis of syntenic gene clusters with this method identified a large number of motifs at p-values below the significance level of FDR < 0.05, including the two motifs identified using the Fisher’s exact test (Table S10).

A total of 65 motifs of 6 nucleotides were found enriched in clusters using the CMH test, as well as 25 motifs of 7 nucleotides and 10 motifs of 8 nucleotides. This result indicated that interspecific analysis using CMH testing appears to increase the power of motif identification over Fisher’s test and in our hands enabled the discovery of a greater number of motifs and longer motifs in more of the co-expression clusters. We applied permutation testing to control the false positive rate through shuffling gene order in the clusters within and between species (Figure S12). The result was lower, significant p-values with shuffling across species compared to shuffling within species for both the CMH test and Fisher’s exact test, suggesting shuffling gene orders across species is more likely to strictly control FDR and result in more statistically reliable motif identification. This CMH analysis identified the CCA1-binding site (CBS, AAAAATCT) and the full EE (AAATATCT) in cluster 2 [51, 54]. Additional *cis*-elements discovered were the morning element (ME, CCACAC) [55] in clusters 1, 11, 12, and 16, as well as the telo-box (TBX, AAACCCT) [56] and starch synthesis box (SBX, AAGCCC) [57] in cluster 5. The SORLIP1 motif (GCCAC) [58, 59] was also identified in clusters 1, 11, 14, and 16. Identification of these *cis*-elements confirmed the power of this motif identification approach. By combining clusters from three species, we found co-expressed genes were more likely to be regulated by conserved motifs, especially the genes with expression phase during the dawn and dark.

## Discussion

### Evolution of circadian clock genes in grass species

Analysis of diurnal gene expression in the three panicoid grasses sorghum, maize, and foxtail millet revealed conserved and divergent evolution of expression for predicted core circadian clock genes. Putative oscillator components these grasses consisted of paralogous gene families in which these paralogs generally showed diurnal expression patterns comparable to their distant *Arabidopsis* orthologs. In a few notable cases, multiple copies of paralogous genes have distinct diurnal expression amplitude and phase patterns, such as paralogs generated by the local WGD in maize. We speculate that multiple paralogous clock genes may have been retained in maize because the individual genes evolved novel roles that allow more accurate differentiation of input signals or specialized regulation of output pathways.

Transcriptome studies have enabled the identification of diurnal gene regulatory networks and circadian clock regulation of key physiological processes [60]. We detected comparable diurnal gene expression shared between sorghum, maize, and foxtail millet with a 72-h time course under diurnal light-dark conditions. We were able to test the hypothesis that syntenic orthologous genes share conserved diurnal expression patterns within these grasses. As expected, the phase of expression for syntenic genes was broadly conserved, but several orthologs had different phases. The potential functional consequences, if any, of these phase differences remains to be determined. A large number of predicted core circadian clock genes, such as members of the *lyl*, *t1l*, *gi*, and *lxl* families, showed matching robust rhythms in all three grasses, which also matched the expression patterns of *Arabidopsis* homologs. This observation is consistent with the general conservation of biological clock mechanism across plants. However, not all of the predicted core circadian clock genes in these grasses had expression patterns comparable to their *Arabidopsis* ortholog. For example, *zll2* and *zll3* were expressed at similar levels and show diurnal rhythms, which is also the case for the sorghum and foxtail millet *zll* orthologs (Figure S7N, O). By contrast, the *Arabidopsis ZTL* gene is not rhythmic [61]. The paralogs of the maize genes, *zll1* and *zll4*, were not expressed, however. Furthermore, the expression phases for the expressed grass *zll* genes was different depending on the gene and the species. *Arabidopsis* ZTL protein is active around the transition from light-dark in the evening [47], but only maize *zll2* had peak expression around at this time (Table 1). Phased nearly 6 h later were the foxtail millet *zll1* and *zll3* genes, along with the sorghum *zll1* gene. The maize and sorghum *zll3* genes were expressed even later, with phases around dawn. An intriguing possibility is the differences in expression amongst of these *zll* genes represents functional divergence for the gene products. We speculate the reason for significantly different expression patterns of these foxtail millet orthologs is related to its temperate origins, short life cycle and/or comparatively early maturation [62, 63], which are attributes different from sorghum and maize.

Previous work indicates that 98% of duplicate genes between maize subgenomes either have divergent expression or have taken on different functions, or subfunctionalized, in a tissue-specific manner [64]. Evidence for both these outcomes is apparent in the diurnal expression behavior of paralogous maize circadian clock genes. One consequence of gene duplication from the maize WGD for circadian clock genes is primarily a dosage effect. The *lyl*, *re7l*, and *p95l* families are cases where both of the duplicated maize genes have lower amplitudes and expression levels compared to the sorghum and foxtail millet orthologs (Figs. 1a, S7E, I), while the *t1l*, *ffl*, *gi*, *re6l*, *ef4r* and *zll* families show one maize copy has an amplitude similar to its orthologs and the other has a much lower amplitude, is expressed at levels too low to detect rhythmicity, or is not expressed at all (Figs. 1b, e-f, S7B-D, K-M). This observation suggests plants maintain gene product balance for economical and efficient metabolism, consistent with the gene balance hypothesis [41]. In other cases, the phase of expression for paralogs resident on the two maize subgenomes is significantly different. For example, the two maize *elfl* genes are expressed 5 h apart, suggesting that the copies could have experienced subfunctionalization.

### Clustering of diurnally-regulated genes across species

Co-expression clustering, which is widely used to mathematically group genes according to the similarity of gene expression patterns [65–68], can greatly enhance our understanding of coordinately expressed genes and their interactive relationships. Functional enrichment of genes in co-expression clusters allows the identification of the contribution of different genes with similar expression patterns to common biological processes [69]. We used a K-means clustering method to identify the shared diurnal co-expression gene network of syntenic orthologs in sorghum, maize, and foxtail millet. Although almost equal numbers of orthologous genes from the three species were clustered and the distribution of the gene phases in each cluster is conserved, only 37.2% of all the syntenic gene groups could be clustered based on diurnal expression pattern. This result is consistent with previous studies in *Arabidopsis* where diurnally expressed genes could be divided into two categories according to potential gene function [69]. The first category represents components of the central clock, while the second category are those genes regulated by the circadian clock. Co-expression clustering provided evidence that diurnal regulation of genes in the first category, such as *LHY*, *CCA1*, *TOC1* and *GI* orthologs, is mostly unaffected by species evolution or external conditions while genes in the second category show considerable divergence in diurnal expression patterns across sorghum, maize, and foxtail millet. While this study focused on conservation of expression patterns, equally interesting would be investigation of genes with non-conserved expression and regulation. Discovery of these genes potentially could indicate evolutionary divergence between the species and pinpoint adaptive divergence.

### Increased power for *cis*-element prediction by combined analysis of syntenic orthologs from multiple species

The behavior of transcriptional regulatory networks is shaped by the activity of conserved DNA sequence motifs in promoter regions. These *cis*-elements are responsible for imparting spatially- and temporally-specific gene expression patterns. Regulatory *cis*-elements have been discovered with single gene-level approaches, like deletion analysis [70], and at the transcriptome-level with computational methods coupled with high-throughput sequencing based on either position weight matrix (PWM) [71, 72] or phylogenetic footprints [73, 74]. The most common approach is to identify motifs in a single species and combine different approaches to increase the reliability of predictions and to decrease the FDR of the prediction [75]. Theoretically, the power of a test could be improved by either increasing the number of genes or the number of species under consideration. Since the number of genes in a given species is fixed, we chose to increase the number of species under consideration. Correspondingly, we clustered syntenic orthologous genes from sorghum, maize, and foxtail millet and identified motifs shared between species under the hypothesis that syntenic orthologous genes with the same expression pattern are likely controlled by the same regulatory elements in each species. We found that Fisher’s exact testing-based methods have limited power to identify motifs in a single species as this approach picked out only two motifs shared between the three grass species.

To improve discovery of statistically significantly enriched *cis*-elements, we adopted the CMH test and this test identified a large number of enriched motifs. This analysis found the CBS and the EE. Both the EE and CBS are binding targets of *Arabidopsis* LHY and CCA1 [51, 54] and contribute to timing of diurnal and circadian gene expression [55, 76]. Additional *cis*-elements involved in timing of diurnal expression discovered were the ME associated with morning expression [55] and the midnight-associated TBX [56] and SBX [57]. The roles of the EE, CBS, ME, TBX, and SBX in temporal organization of diurnal expression modules are conserved across *Arabidopsis*, rice, poplar, and papaya [22, 77]. The SORLIP1 motif is enriched in light-induced genes in *Arabidopsis* and rice [58, 59]. Discovery of well-known motifs indicates the CMH approach has the power to identify actual *cis*-elements, which suggests the other conserved sites identified are authentic but currently unknown motifs. These predictions remain to be confirmed with functional tests.

Computational identification of *cis*-elements has the shortcoming of a high false-positive rate. There were several methods to control the FDR, such as retaining the motifs conserved in combining multiple approaches, choosing the motifs associated with at least two genes to increase their likelihood to be actual functional *cis*-motifs, and detecting the higher appearance frequency of the motifs acting as binding sites of an established transcription factor [75]. Our analysis controlled the FDR in three ways. The first method was utilizing the conservation of DNA sequences adjacent to orthologous genes across species, given the assumption that genes with similar expression patterns are likely to share common regulatory mechanism. The identification of motifs associated with genes having diurnal expression patterns was based on sequence conservation of motifs shared among sorghum, maize and foxtail millet. The second method served as a negative control, which consisted of permutation test based on shuffling the distribution of genes in the clusters across species. The third was the use of K-means clustering to identify syntenic orthologs with the same expression pattern since the resultant clusters are the output of shared gene regulatory networks representing conserved regulatory mechanisms. Based on this analysis, we conclude that patterns of diurnal gene expression are highly conserved and the architecture of these diurnal regulatory networks relies on conserved local regulatory sequences.

## Conclusions

The circadian clock drives endogenous 24-h rhythms that allow organisms to adapt and prepare for predictable and repeated changes in their environment throughout the diurnal cycle. Circadian clock components in sorghum, maize, and foxtail millet consist of syntenic orthologous gene families that generally have diurnal expression patterns comparable to distant *Arabidopsis* orthologs. Notably, most paralogs in maize generated by the local WGD exhibit clear differences between each other in either expression amplitude and/or phase. We predict each paralogous gene may have taken on a separate role, potentially to allow more accurate differentiation of input signals or for specialized regulation of output pathways. Co-expression analysis identified well-known motifs and novel DNA sequence motifs predicted to be regulatory *cis*-elements that shape diurnal expression patterns for genes involved in common physiological activities. We conclude that patterns of diurnal gene expression are highly conserved and conserved local regulatory sequences contribute to the architecture of diurnal regulatory networks in pancoid grasses.

## Methods

### Plant material and growth conditions

Plants for tissue sampling were grown from seeds of reference genotypes in *Zea mays* (genotype B73), *Sorghum bicolor* (genotype BTx623) and *Setaria italica* (genotype Yugu1). All of the seedlings were grown in parallel in a common walk-in growth chamber at the Plant Gene Expression Center. Growth conditions were 12 h light and 12 h dark and daytime temperatures were between 26 and 28 *°*C and nighttime temperature was 22 *°*C. Light was provided by cool white fluorescent bulbs with 440 μmol m^− 1^ s^− 1^ of photosynthetically active radiation (PAR) at shelf level. Plants of each species were grown to the 3 leaf stage, which is when the third leaf is fully expanded and has a clear ligule and auricle. Representative plants are shown in Figure S1. Once plants reached this developmental benchmark, entire third leaf blades were collected from multiple plants (2 leaves for sorghum and 4 leaves for maize and foxtail millet), pooled and immediately frozen in liquid nitrogen. Samples were taken every 3 hours from a new set of plants over 72 h (8 time points per day, 24 total time points per species). Total RNA was extracted with TRIzol reagent (Thermo Fisher Scientific) according to the manufacturer’s recommendations and RNA libraries were constructed as described by Wang et al. [78]. Illumina HiSeq 2500 sequencing was conducted at the Illumina Sequencing Genomics Resources Core Facility at Weill Cornell Medical College. Quality control of the raw reads were conducted using Cutadapt v1.10 [79] and reads were aligned to corresponding genomes (sorghum: v3.1; foxtail millet; v2.2; maize: 5b) using Gmap/Gsnap [80] and Samtools [81]. Expression level for individual transcripts were computed as FPKM, which were calculated using Cufflinks v2.2.1 [82].

### Construction of protein phylogenetic trees for core circadian genes

We combined literature curation and publicly available databases to search for putative homologs of *Arabidopsis* circadian clock genes in well-sequenced eudicot and monocot species, including soybean, tomato, poplar, *Brassica rapa*, banana, rice, *Brachypodium distachyon*, foxtail millet, sorghum, maize, *Amborella trichopoda* and *Selaginella moellendorffii*. We began with a protein list of five known types of circadian clock genes from *Arabidopsis* (https://www.arabidopsis.org) that was constructed based on the gene functions in the MaizeGDB (https://maizegdb.org/), Phytozome (https://phytozome.jgi.doe.gov/), and information in the literature (Table S5). NCBI protein BLAST (BLASTp; https://blast.ncbi.nlm.nih.gov/Blast.cgi) with default parameters was used for alignment and the top BLASTp hits by coverage and e-value were chosen. The protein sequences of other plant species were obtained from UniProt (https://www.uniprot.org/) after identifying the target protein in MaizeGDB or Phytozome using the chromosomal location provided by the GeneID listing. Protein sequences were used for phylogenetic tree construction rather than nucleotide sequences because of the long evolutionary distances between species. Sequence alignments were performed using the MAFFT v7.450 [83] online tool (https://mafft.cbrc.jp/alignment/server) in G-INS-1 (progressive method with an accurate guide tree) with an Unalignlevel = 0.4, and maximum likelihood phylogenetic trees were built using RAxML-IV-HPC v8.2.12 [84] on the CIPRES server [85] (https://embnet.vital-it.ch/raxml-bb/). Homologs in multiple species analyzed were named according to maize nomenclature rules, which specify the maize genes in lower-case names and paralogs are denoted with numbers (https://www.maizegdb.org/nomenclature). Genes located on maize subgenomes were numbered as 1 or 2 for subgenome maize1 and subgenome maize2, respectively. We retained gene names for published homologous genes. Previous working names of genes, as well as their maize subgenome are listed in Table S6. Protein domains were identified using the Prosite (https://prosite.expasy.org/) and Pfam (https://pfam.xfam.org/).

### Identification of rhythmic genes in time series gene expression dataset

Genes with rhythmic expression were identified and their phase, amplitude and period were estimated with the non-parametric JTK_Cycle algorithm with default settings [86]. JTK_Cycle identifies the optimal period and phase to minimize the p-value representing the correlation between an experimental time series and cosine curve-derived models. The p-value is then Bonferroni-adjusted for multiple testing. JTK_Cycle analysis was done with the full 72-h time course for each gene having detectable expression at one or more of the 24 time points. This approach is the optimal experimental design for identification of rhythmic gene expression patterns according to comprehensive testing of published approaches for rhythmic gene discovery [87]. Rhythmic genes were considered those with Bonferroni-adjusted p-values ≤0.01, which corresponds to a false positive rate of 2 and a false negative rate of 9 [86]. Phase values (LAG in the JTK_Cycle output) were adjusted to circadian time (CT) with the calculation: CT phase = (JTK_Cycle LAG/estimated period) * 24. CT0 is equal to 9:00 AM.

### K-means clustering and gene expression pattern comparisons

The list of syntenic genes from sorghum, maize, and foxtail millet used has been publicly released online [49] and was generated using QuotaAlign [88] followed by ortholog assignment polishing as described in [89] (Table S7). A total of 57,802 genes from 17,744 syntenic orthologous groups in the three species were selected based on the criterion of detectable expression at one or more of the 24 time points. A gene expression matrix was generated from this gene set. Genes with low expression reproducibility across the 72-h time course were removed from the further analysis. Gene expression reproducibility was determined by calculating the Pearson correlation coefficient between pairwise 21-h temporal windows (time points 1–8, 9–16, 17–24) within the three species groups and dispersion determined by MSD among these temporal windows (Figure S8A). Since these two filter parameters had the potential to reduce the number of remaining genes and we expected syntenic genes with similar expression patterns to be appear in the same K-means cluster, we tested the remaining total number of genes and the number of paired syntenic genes in the same cluster for all the combinations of Pearson correlation coefficients ranging from 0.1 to 0.9, with step length by 0.1, and MSD ranging from 0.1 to 0.9, with step length by 0.1 (Figure S8). Based on this analysis, we retained gene groups having a Pearson correlation between two windows higher than 0.7 and mean signed deviation (MSD) lower than 0.9, resulting in 27,196 total orthologous genes for clustering (8616 sorghum genes, 8836 foxtail millet genes, and 9744 maize genes).

Furthermore, it was possible that syntenic gene pairs would cluster together by chance without sharing the same expression pattern. To study the background distribution of syntenic gene pairs, we applied a permutation test that consisted of shuffling the assignment of syntenic genes to clusters and calculating the percentage of clustered syntenic gene pairs (Figure S9). In this permutation test, we checked only syntenic gene pairs between sorghum and foxtail millet to avoid complications from maize genes arising from duplication during the local WGD event in the maize lineage. K-means clustering was performed for 2 to 24 centers to group the syntenic genes based on their expression values using Pearson correlation with 2000 iterations (Figure S9). This permutation test indicated that 16 clusters was the optimal number of K-means centers, since this value produced clusters with the greatest number of sorghum-foxtail millet syntenic gene pairs and the lowest false positive cases.

### Functional analysis of genes in co-expressed clusters

GOATOOLS v0.5.9 [90] was used to associate co-expressed genes with Gene Ontology (GO) categories to identify functional enrichment for genes in co-expression clusters. GO enrichment analysis was performed on the genes of each species in the 16 clusters using the Bonferroni correction to control the significance (p-value: *<* 0.05), with the total number of genes equal to the complete syntenic gene set.

### *cis*-element identification in sorghum, maize, and foxtail millet

Random sequences of 6 to 8 nucleotides were used to test for enrichment of motifs upstream of genes in co-expression clusters. For motifs of each length, the 1 kilobase predicted promoter region upstream of the transcription start site was scanned for all permutations of a sequence for each species. Counting included the number of motifs discovered in each cluster and in all clusters together. Fisher’s exact testing was applied to test enrichment of each possible sequence for a single species. The CMH test was employed to test the combined multiple 2 × 2 tables of all three species and to calculate a combined p-value. The p-value in the CMH test is expected to be more stringent than any one individual p-value if a real connection existed across all three species. The permutation test was run 100 times to provide an adjusted p-value to test the significance. The motifs in both tests with the false discover rate (FDR) p-value < 0.05 were considered high quality motif predictions and these motifs were matched to known *cis*-element DNA sequences through literature curation and the PLACE database (https://www.dna.affrc.go.jp/PLACE) [91].

## Supplementary information

**Additional file 1: Figure S1.** Representative plants at the time of sampling for RNA-seq. **Figure S2.** Phase distribution of all genes called rhythmic by JTK-Cycle analysis. **Figure S3.** Amplitude distribution of genes called rhythmic by JTK_Cycle analysis. **Figure S4.** Large protein trees constructed for the RVE/LHY-like, PRR, and ELF4 protein families. **Figure S5.** Maximum likelihood phylogenetic tree of LYL protein family. **Figure S6.** Maximum likelihood phylogenetic trees of ELF4 and EF4L protein families. **Figure S7.** Diurnal expression of circadian clock and clock-associated orthologs of over the 72-h time course. **Figure S8.** Heatmaps of feature statistics under different filter settings of Pearson correlation (Cor) and mean signed deviation (SD). **Figure S9.** Effect of center number on clustering of syntenic genes by K-means. **Figure S10.** Comparison of phase distribution for orthologous maize and sorghum genes with diurnal expression. **Figure S11.** Comparison of the phase distribution for orthologous maize and foxtail millet genes with diurnal expression. **Figure S12.** Comparison of gene shuffling methods for the permutation test.

**Additional file 2: Table S1.** Summary of RNA-seq information for maize, sorghum, and foxtail millet. **Table S2.** Rhythmic genes in sorghum. **Table S3.** Rhythmic genes in maize. **Table S4.** Rhythmic genes in foxtail millet. **Table S5.** Circadian clock and clock-associated proteins from *Arabidopsis*. **Table S6.** Circadian clock and clock-associated proteins from maize and orthologs from sorghum and foxtail millet. **Table S7.** Syntenic orthologous genes from sorghum, maize, and foxtail millet. **Table S8.** Gene composition of K-means co-expression clusters. **Table S9.** GO categories enriched in K-means co-expression clusters. **Table S10.** Statistically enriched motifs upstream of genes in K-means co-expression clusters.

## Data Availability

The datasets generated during the current study are available as a BioProject at the National Center for Biotechnology Information under accession number PRJNA616061 (https://dataview.ncbi.nlm.nih.gov/object/PRJNA616061). Arabidopsis diurnal gene expression microarray datasets shown in Table 1 from reference [37] are available at ArrayExpress under accession number E-MEXP-1304 (https://www.ebi.ac.uk/arrayexpress/experiments/E-MEXP-1304/). DNA sequences corresponding to *cis*-regulatory motifs were obtained from the PLACE database version 30.0 (files place.dat (https://www.dna.affrc.go.jp/PLACE/place_dat.shtml) and place.seq (https://www.dna.affrc.go.jp/PLACE/place_seq.shtml)). Protein amino acid sequences were obtained from Uniprot release 2015.01 (ftp://ftp.uniprot.org/pub/databases/uniprot/previous_releases/release-2015_01/). Protein domains were predicted based on Prosite version 6.1 (ftp://ftp.expasy.org/databases/prosite/old_releases/prosite06_1.tar.bz2) and Pfam version 9.0 (ftp://ftp.ebi.ac.uk/pub/databases/Pfam/releases/Pfam9.0/). Arabidopsis genes/proteins from The Arabidopsis Information Resource (https://www.arabidopsis.org), annotation version TAIR10 (name (unique gene id; Uniprot accession number)): LHY (AT1G01060; Q6R0H1), CCA1 (AT2G46830; P92973), RVE1 (AT5G17300; F4KGY6), RVE2 (AT5G37260; F4K5X6), RVE4 (AT5G02840; Q6R0G4), RVE5 (AT4G01280; C0SVG5), RVE6 (AT5G52660; Q8H0W3), RVE7 (AT1G18330; B3H5A8), RVE8 (AT3G09600; Q8RWU3), TOC1/PRR1 (AT5G61380; Q9LKL2), PRR3 (AT5G60100; F4JXG7), PRR4 (AT5G49240; Q9FJ16), PRR5 (AT5G24470; Q6LA42), PRR7 (AT5G02810; Q93WK5), PRR9 (AT2G46790; Q8L500), GI (AT1G22770; Q9SQI2), ELF3 (AT2G25930; O82804), ELF4 (AT2G40080; O04211), EF4L1 (AT2G29950; O80877), EF4L2 (AT1G72630; Q94BS8), EF4L3 (AT2G06255; Q8S8F5), EF4L4 (AT1G17455; Q570U6), LUX)/PCL1 (AT3G46640; F4J959), BOA (AT5G59570; F4J959), ZTL/ADO1 (AT5G57360; F4KAN2), LKP2/ADO2 (AT2G18915; Q8W420), FKF1/ADO3 (AT1G68050; Q9C9W9). Genes/proteins from MaizeGDB (https://maizegdb.org/) for maize annotation version 5b, and from Phytozome (https://phytozome.jgi.doe.gov/) for sorghum annotation version v3.1 and foxtail millet annotation version v2.2 (name (maize; sorghum; foxtail millet unique gene id)): LYL1 (GRMZM2G175227 + GRMZM2G175265 + GRMZM2G474769; Sobic.007G047400; Seita.6G055700), LYL2 (GRMZM2G014902; Sobic.007G047400; Seita.6G055700), RE2L (GRMZM2G145041; Sobic.010G275700; Seita.4G288100), RE6L1 (GRMZM2G135052; Sobic.010G004300; Seita.4G004600), RE6L2 (GRMZM2G170148; Sobic.010G223700; Seita.4G266800), RE6L3 (GRMZM2G057408; Sobic.010G223700; Seita.4G266800), RE6L4 (GRMZM5G833032; Sobic.004G281800; Seita.1G272700), RE6L5 (GRMZM2G118693; Sobic.004G281800; Seita.1G272700), RE7L1 (GRMZM2G029850, Sobic.004G279300; Seita.1G275400), RE7L2 (GRMZM2G170322; Sobic.004G279300; Seita.1G275400), RE7L3 (GRMZM2G421256; Sobic.006G192100; Seita.7G212900), RE7L4 (GRMZM2G181030; Sobic.006G192100; Seita.7G212900), RE8L2 (GRMZM2G115070; Sobic.001G143100, Si038786m); RE8L1 (GRMZM2G415077; Sobic.001G143100; Si038786m), T1L1 (GRMZM2G148453; Sobic.004G216700; Seita.1G236100), T1L2 (GRMZM2G020081; Sobic.004G216700; Seita.1G236100), P37L1 (GRMZM2G005732; Sobic.006G057900; Seita.2G444300), P37L2 (GRMZM2G033962; Sobic.006G057900; Seita.2G444300), P59L2 (GRMZM2G488465 + GRMZM2G013913; Sobic.005G044400; Seita.8G040100), P59L1 (GRMZM2G135446; Sobic.005G044400; Seita.8G040100), P73L (GRMZM2G095727; Sobic.001G411400; Seita.9G445200), P95L1 (GRMZM2G179024; Sobic.002G275100 Seita.2G286100), P95L2 (GRMZM2G367834; Sobic.002G275100; Seita.2G286100), GI1 (GRMZM2G107101; Sobic.003G040900; Seita.5G129500), GI2 (GRMZM5G844173; Sobic.003G040900; Seita.5G129500), EF3L1 (GRMZM2G045275; Sobic.003G191700; Seita.5G204600), EF3L2 (AC233870.1_FG003; Sobic.009G257300; Seita.3G121000), EF4R1 (GRMZM2G382774; Sobic.001G340700; Seita.9G368100), EF4R2 (GRMZM2G359322; Sobic.001G340700; Seita.9G368100), EF4R3 (GRMZM5G877647; Sobic.005G194200; Seita.8G200900), EF4R4 (GRMZM2G025646, Sobic.002G193000; Seita.2G195800), LXL (GRMZM2G067702; Sobic.003G443600; Seita.5G468100), ZLL1 (GRMZM2G115914; Sobic.010G243900; Seita.4G249100), ZLL2 (GRMZM2G113244; Sobic.010G243900; Seita.4G249100), ZLL3 (GRMZM2G147800; Sobic.004G042200; Seita.1G087300), ZLL4 (GRMZM2G166147; Sobic.004G042200; Seita.1G087300), FFL1 (GRMZM2G106363, Sobic.005G145300; Seita.8G146900), FFL2 (GRMZM2G107945; Sobic.005G145300; Seita.8G146900).
