# Supplementary material for: Interspecific analysis of diurnal gene regulation in panicoid grasses identifies known and novel regulatory motifs
Source: BMC Genomics. 2020 Jun 25;21:428. doi: 10.1186/s12864-020-06824-3 (PMC7315539; doi:10.1186/s12864-020-06824-3)
Supplement: Supplementary file 1 — Additional file 1: Figure S1. Representative plants at the time of sampling for RNA-seq. Figure S2. Phase distribution of all genes called rhythmic by JTK-Cycle analysis. Figure S3. Amplitude distribution of genes called rhythmic by JTK_Cycle analysis. Figure S4. Large protein trees constructed for the RVE/LHY-like, PRR, and ELF4 protein families. Figure S5. Maximum likelihood phylogenetic tree of LYL protein family. Figure S6. Maximum likelihood phylogenetic trees of ELF4 and EF4L protein families. Figure S7. Diurnal expression of circadian clock and clock-associated orthologs of over the 72-h time course. Figure S8. Heatmaps of feature statistics under different filter settings of Pearson correlation (Cor) and mean signed deviation (SD). Figure S9. Effect of center number on clustering of syntenic genes by K-means. Figure S10. Comparison of phase distribution for orthologous maize and sorghum genes with diurnal expression. Figure S11. Comparison of the phase distribution for orthologous maize and foxtail millet genes with diurnal expression. Figure S12. Comparison of gene shuffling methods for the permutation test. [file 12864_2020_6824_MOESM1_ESM.docx]

**SUPPLEMENTARY FIGURES**

**
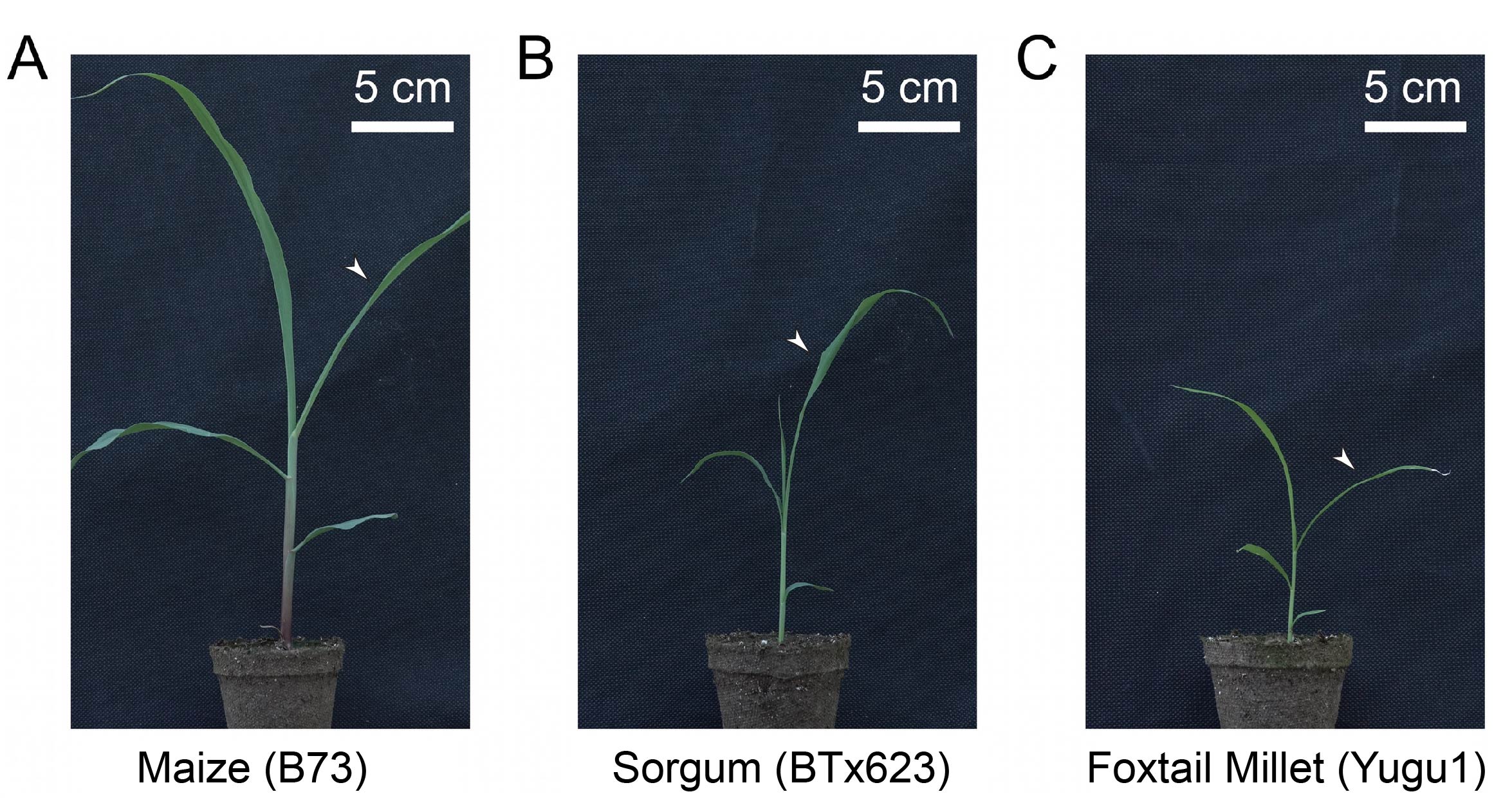
**

**Figure S1.** Representative plants at the time of sampling for RNA-seq. Maize (A), sorghum (B), and foxtail millet (C) plants at the stage that leaf 3 (indicated by white arrow) was sampled for gene expression. Images of plants were taken prior to sampling.


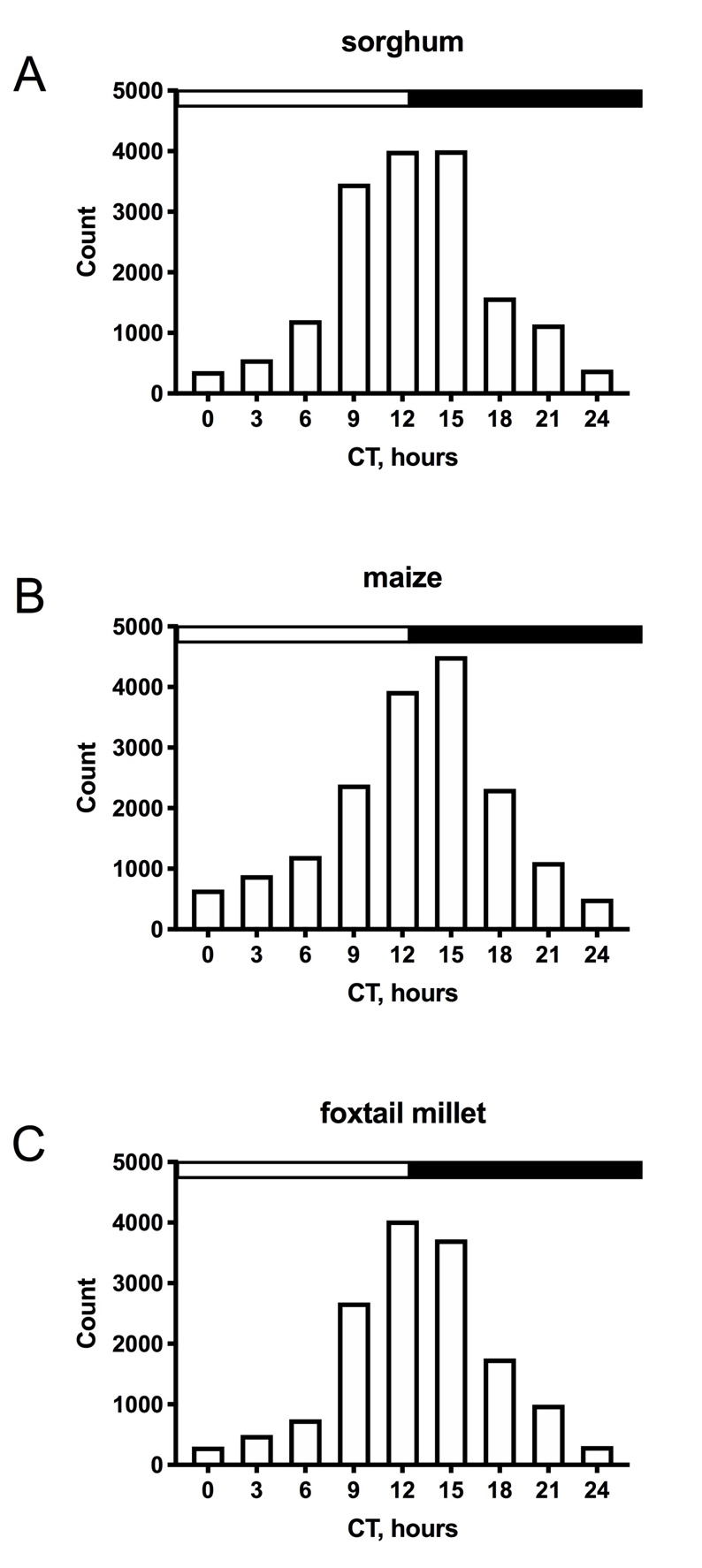


**Figure S2.** Phase distribution of all genes called rhythmic by JTK_Cycle analysis. The y-axis shows gene counts for maize (A), sorghum (B), and foxtail millet (C). The x-axis shows the predicted CT-corrected phase of each gene. White and black bars correspond to times of light and dark, respectively.

**Figure S3.** Amplitude distribution of genes called rhythmic by JTK_Cycle analysis. Amplitude values are for genes falling within the 90th percentile of amplitudes for all rhythmic genes.


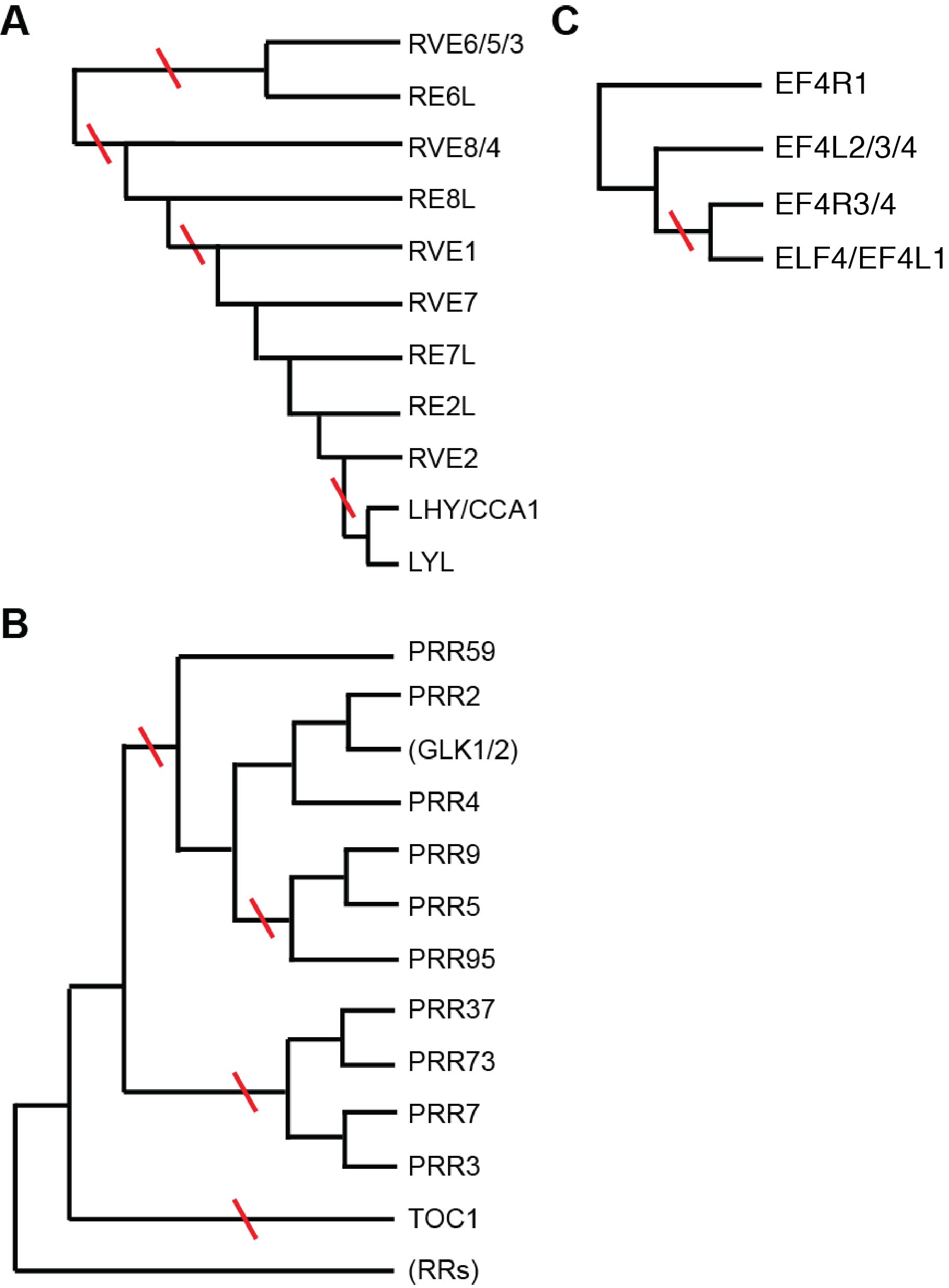


**Figure S4.** Large protein trees constructed for the RVE/LHY-like, PRR, and ELF4 protein families. Subtrees were extracted from the larger tree where indicated by the red slashes. (A) The RVE/LHY-like protein family has four subtrees: the RVE6 group, the RVE8 group, the LHY-like group and the group encompassing RVE1, RVE7, and RVE2. (B) The PRR protein family has four subtrees: the PRR9/5/95 groups, the PRR3/7/37/73 group, the TOC1group, and the group with PRR59 and PRR2/4. Proteins in the PRR2/4 family are not circadian clock-associated. Parentheses around groups GLK (GOLDEN2-LIKE) and RRs (response regulators) indicate they were not included in the subtree. (C) The ELF4 protein family has two subtrees: the ELF4/ELF4L1 and EFR3/4 groups, and the ELFL2/3/4 and E4R1 groups.


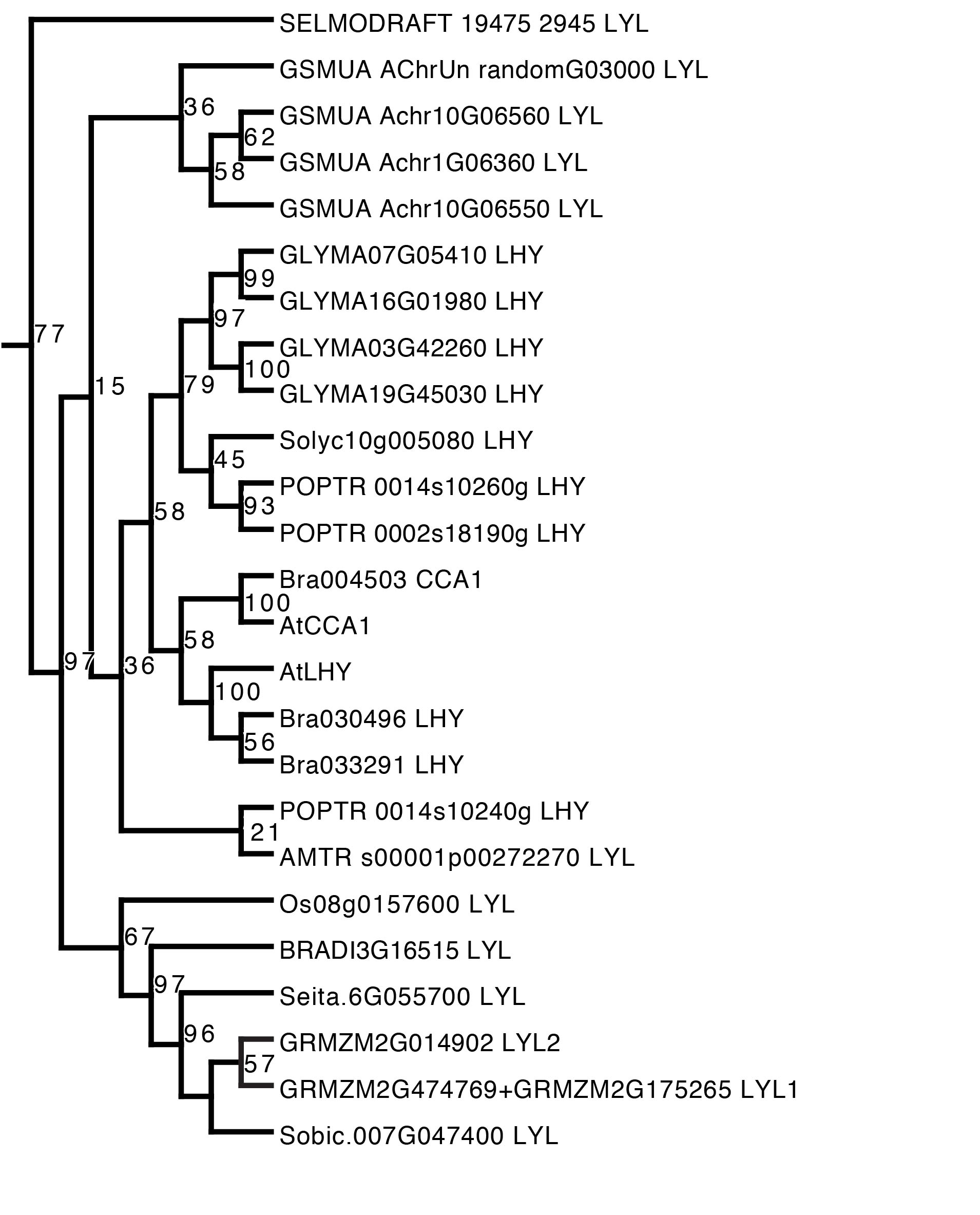


**Figure S5.** Maximum likelihood phylogenetic tree of LYL protein family. Numbers indicate percentage bootstrap values from 1000 rapid bootstrap inferences. Proteins are from *Amborella trichopoda* (AMTR), soybean (GLYMA), tomato (Solyc), poplar (POPTR), *Brassica rapa* (Bra), banana (GSMUA), rice (Os), *Brachypodium distachyon* (BRADI), foxtail millet (Si), sorghum (Sb), maize (GRMZM), and *Selaginella moellendorffii* (SELMODRAFT).


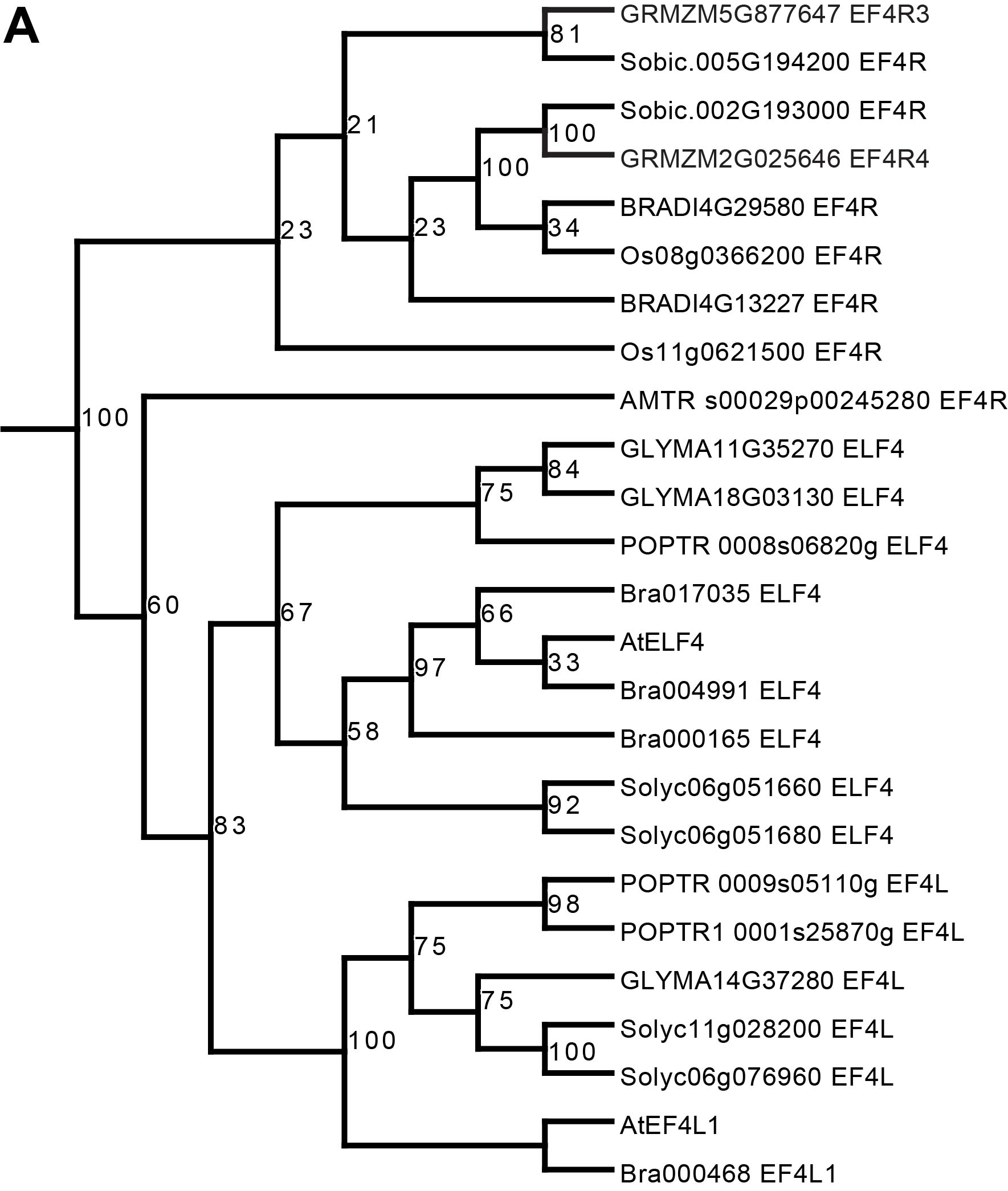


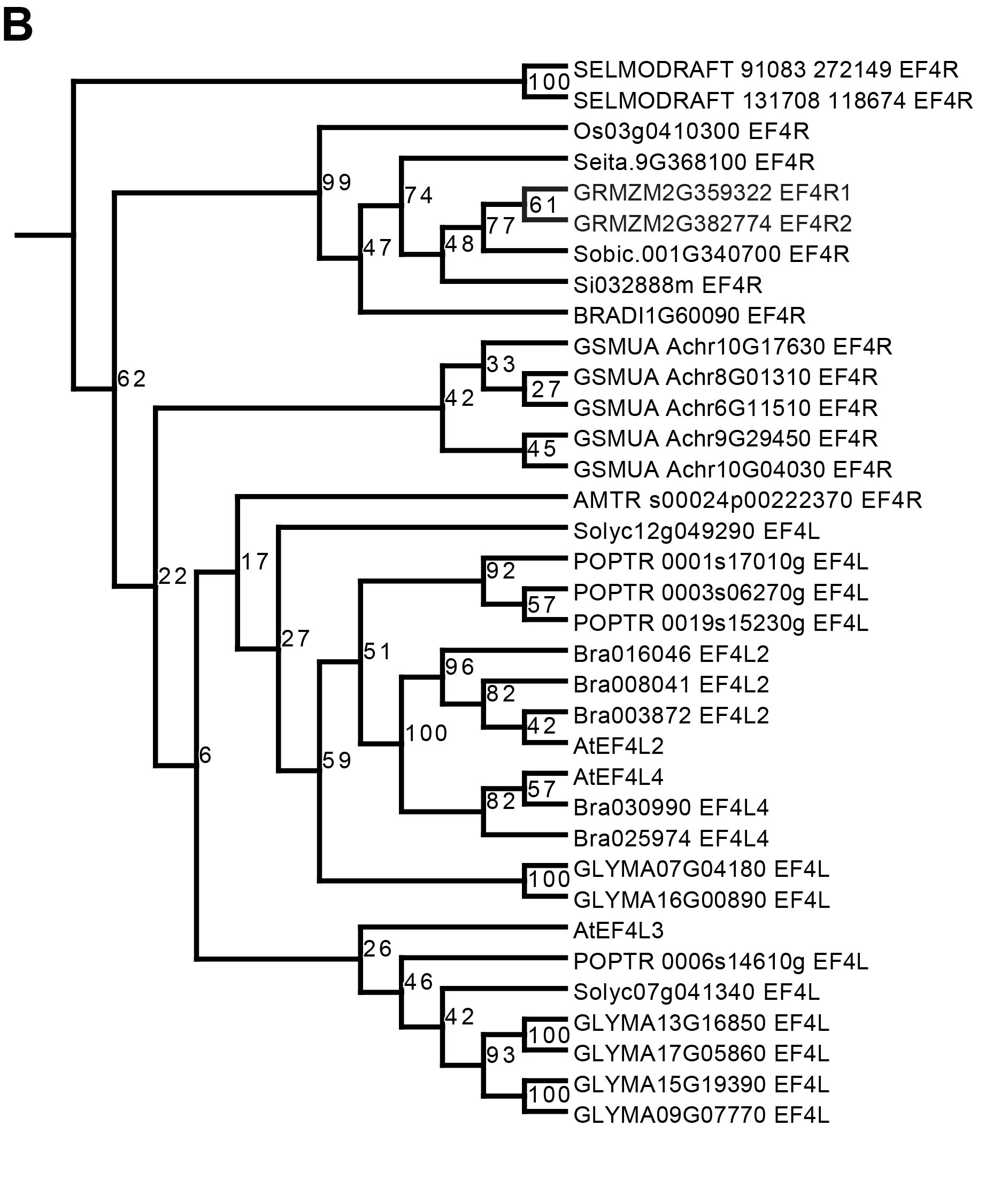


**Figure S6.** Maximum likelihood phylogenetic trees of ELF4 and EF4L protein families. (A) Tree of the ELF4/ELF4L1 and EFR3/4 groups. (B) Tree of the ELFL2/3/4 and E4R1/2 groups. Numbers indicate percentage bootstrap values from 1000 rapid bootstrap inferences. Proteins are from *Amborella trichopoda* (AMTR), soybean (GLYMA), tomato (Solyc), poplar (POPTR), *Brassica rapa* (Bra), banana (GSMUA), rice (Os), *Brachypodium distachyon* (BRADI), foxtail millet (Si), sorghum (Sb), maize (GRMZM), and *Selaginella moellendorffii* (SELMODRAFT).


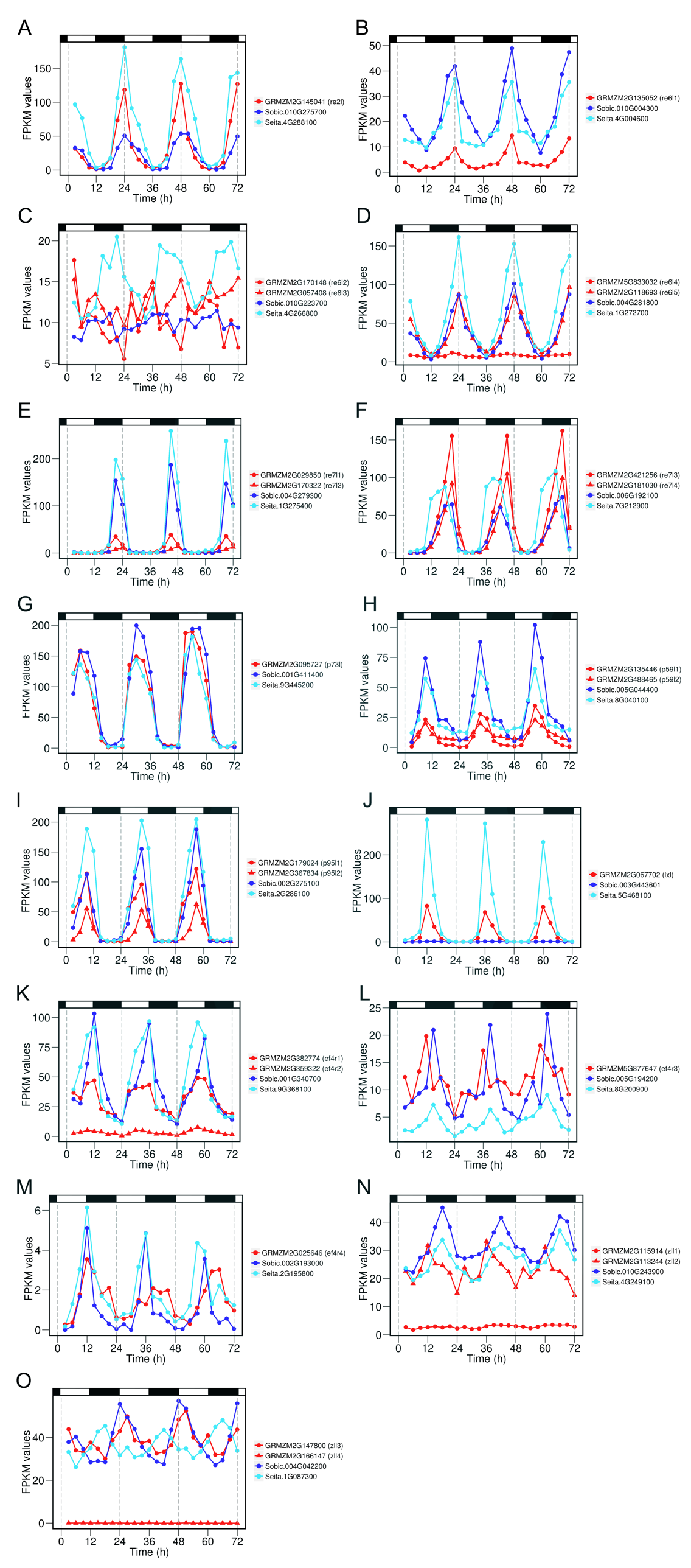


**Figure S7.** Diurnal expression of circadian clock and clock-associated orthologs of over the 72-hour time course. Expression levels of (A) *re2l*, (B) *re6l1*, (C) *re6l2/3*, (D) *re6l4/5*, (E) *re7l1/2*, (F) *re7l3/4*, (G) *p73l*, (H) *p59l1/2*, (I) *p95l1/2*, (J) *lxl*, (K) *zll1/2*, and (L) *zll3/4*.

FPKM values shown for maize (red), sorghum (blue), and foxtail millet (light blue). White and black bars correspond to times of light and dark, respectively.


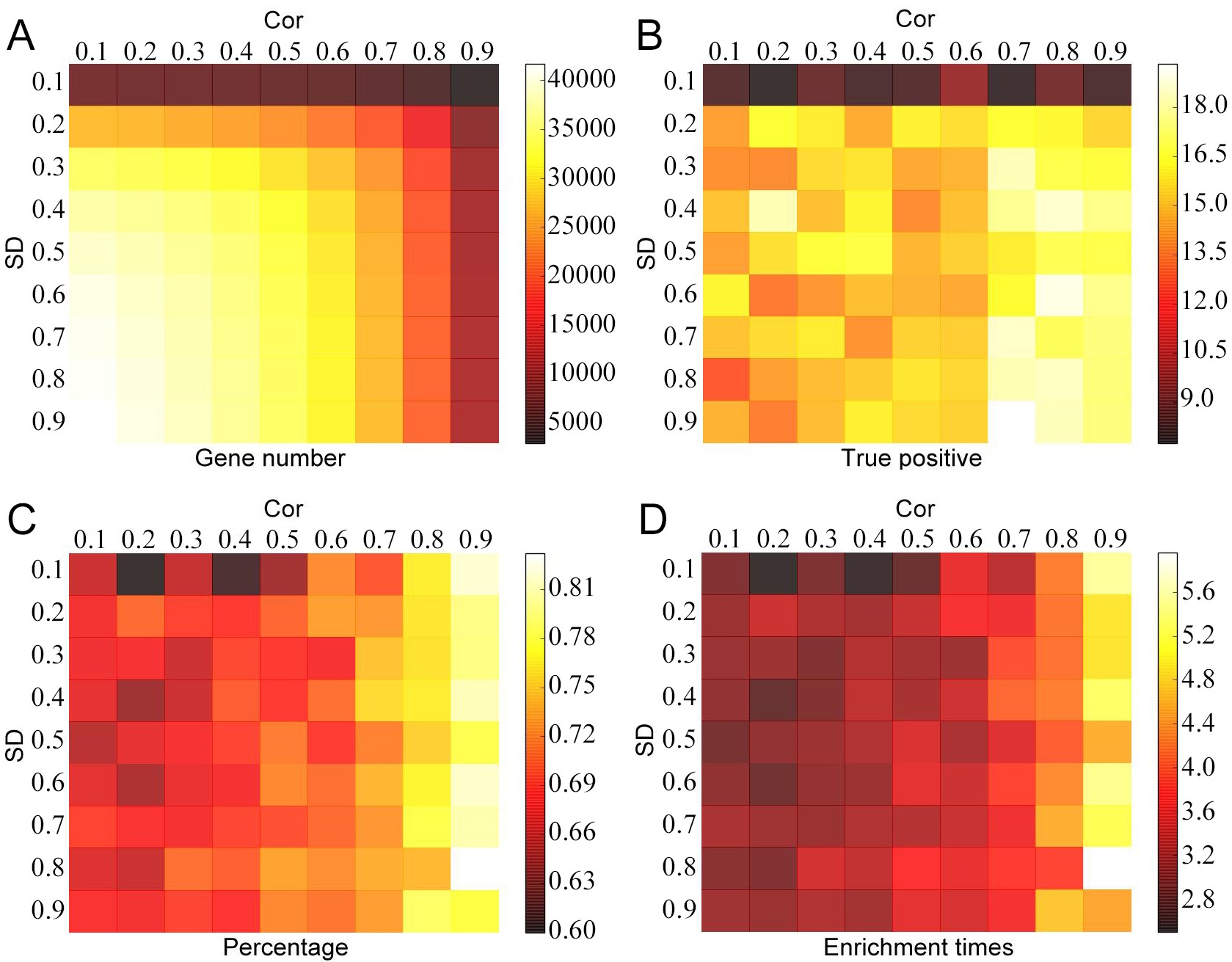


**Figure S8.** Heatmaps of feature statistics under different filter settings of Pearson correlation (Cor) and mean signed deviation (SD). Permutation tests were performed 100 times with the same parameters and median values were regarded as the false positives. (A) Number of genes passing the filter. (B) True positive proportion of sorghum-foxtail millet syntenic genes staying in the same clusters. (C) Percentage of true positives divided by the total syntenic genes in the same cluster. (D) Times of true syntenic values over the median permutation.


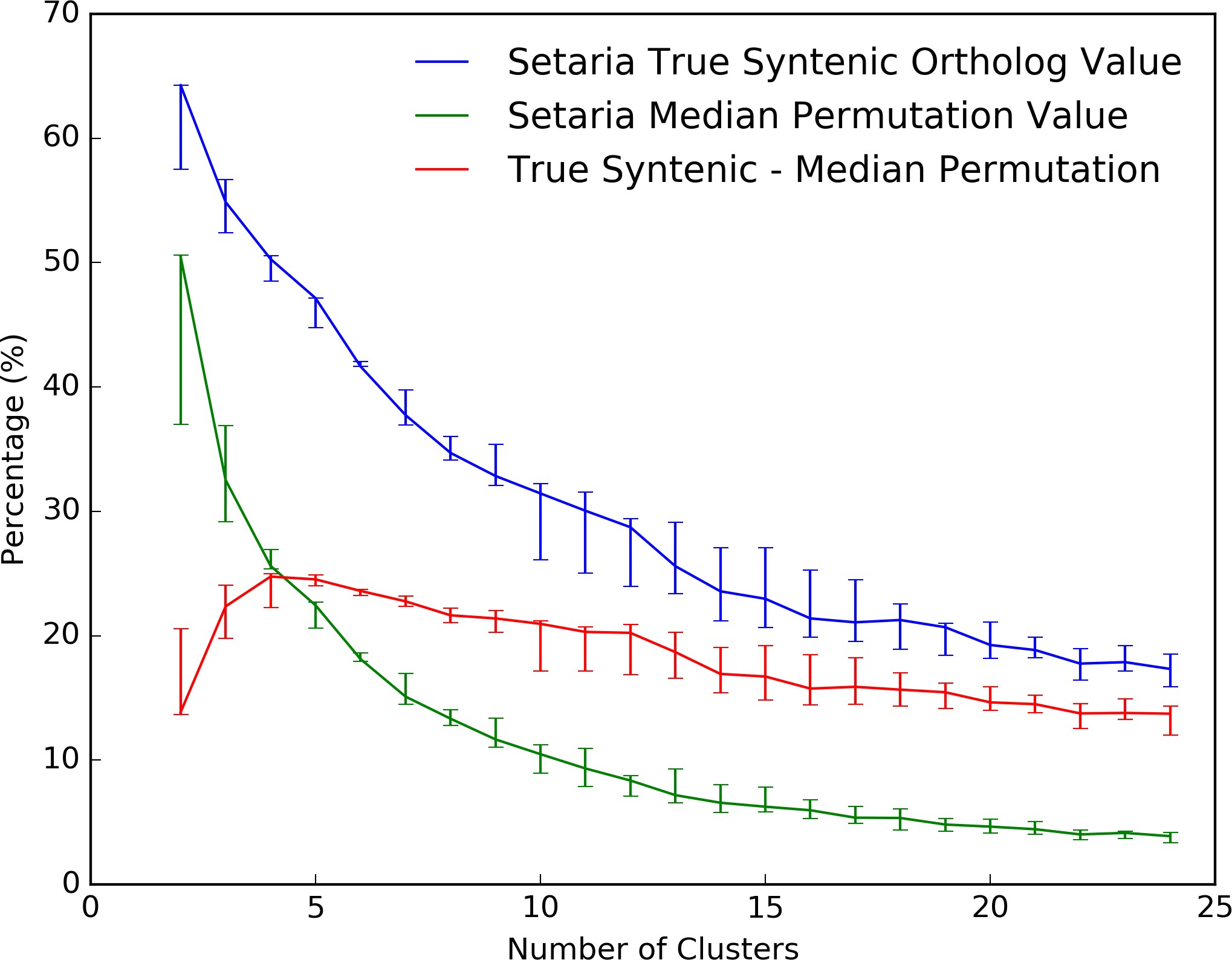


**Figure S9.** Effect of center number on clustering of syntenic genes by K-means. The x-axis shows the number of centers in each clustering test. The y-axis shows the percentage of sorghum-foxtail millet syntenic genes grouped in the same clusters, corresponding to the true positive ratio. The error bars indicate the range of percentage in 10 replicates.


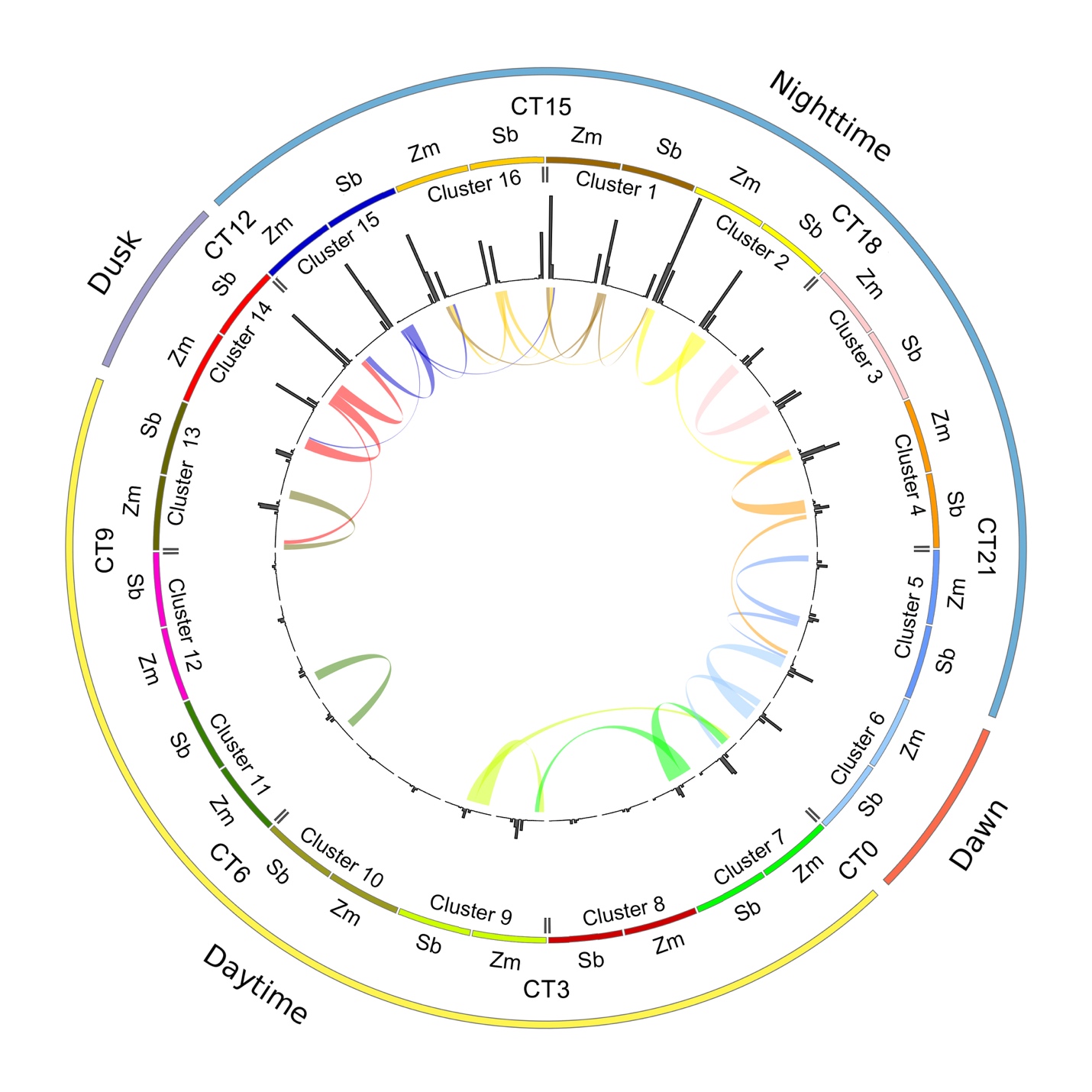


**Figure S10.** Comparison of phase distribution for orthologous maize and sorghum genes with diurnal expression. Concentric circles represent different features layered together from the outside to inside. The outer layer represents dawn (red), daytime (yellow), dusk (purple), and nighttime (blue) and the corresponding CT for these parts of the diurnal cycle. CT0 is equal to 9:00 AM. The second layer shows the relative temporal position of the median gene expression for maize and sorghum orthologs in the 16 co-expression clusters, where each color represents one cluster. The third layer shows the 24-hour temporal distribution of peak expression for genes in that cluster along a line beginning and ending at CT12 (midnight). The inner most layer shows the cluster position of enriched syntenic genes shared between maize and sorghum based on the null model. Each colored line represents at least 10 shared genes and line thickness is proportional to the number of shared genes.


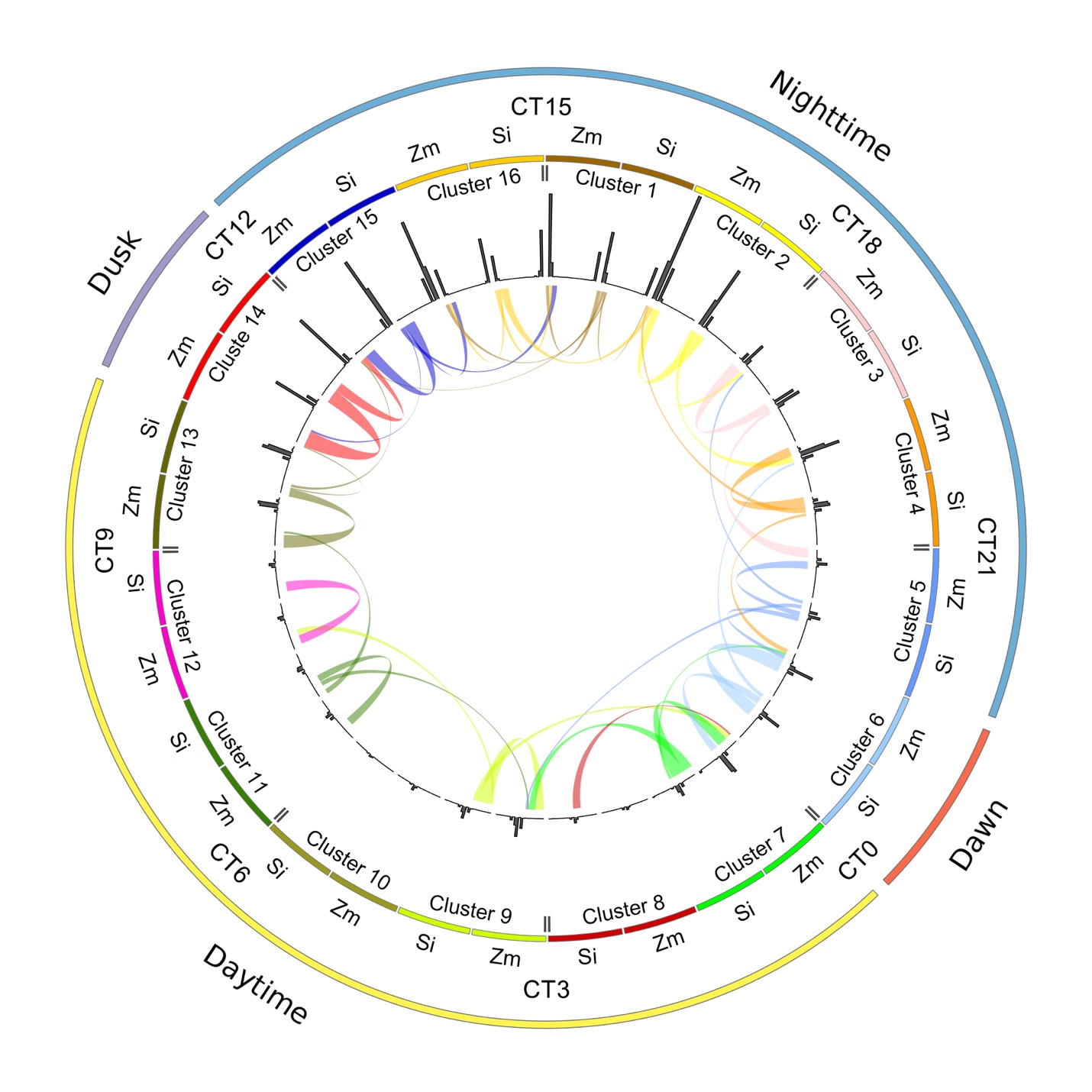


**Figure S11.** Comparison of phase distribution for orthologous maize and foxtail millet genes with diurnal expression. Concentric circles represent different features layered together from the outside to inside. The outer layer represents dawn (red), daytime (yellow), dusk (purple), and nighttime (blue) and the corresponding CT for these parts of the diurnal cycle. CT0 is equal to 9:00 AM. The second layer shows the relative temporal position of the median gene expression for maize and foxtail millet orthologs in the 16 co-expression clusters, where each color represents one cluster. The third layer shows the 24-hour temporal distribution of peak expression for genes in that cluster along a line beginning and ending at CT12 (midnight). The inner most layer shows the cluster position of enriched syntenic genes shared between maize and foxtail millet based on the null model. Each colored line represents at least 10 shared genes and line thickness is proportional to the number of shared genes.


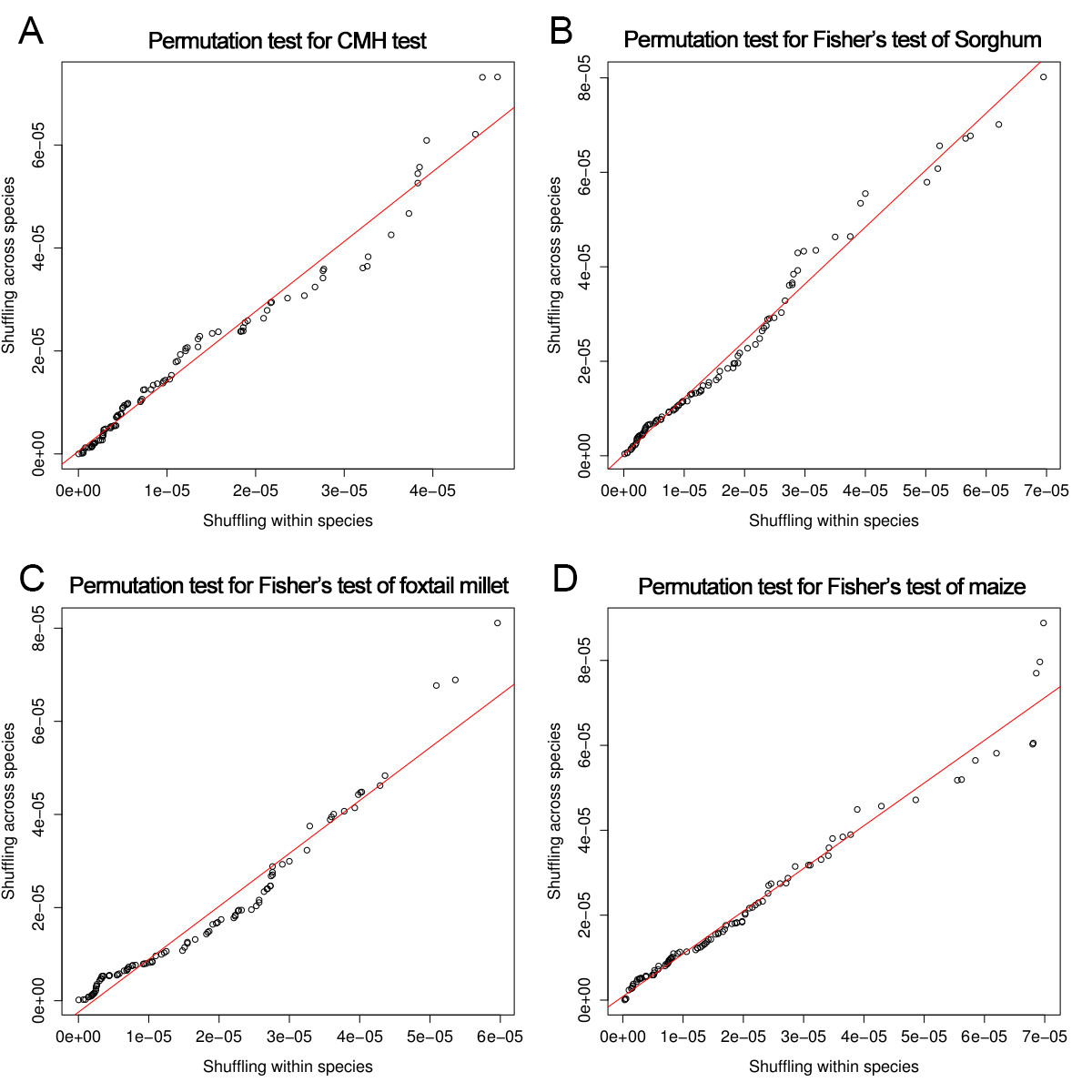


**Figure S12.** Comparison of gene shuffling methods for the permutation test. The x-axis shows the permutation test by shuffling the genes within species and y-axis shows the permutation test by shuffling the genes across species. (A) The differences between two gene shuffling methods in Cochran–Mantel–Haenszel (CMH) test. (B), (C) and (D) the differences between two gene shuffling methods on Fisher’s exact test t for sorghum, foxtail millet, and maize. The permutation was performed 100 times and the top significant value of each permutation is shown. The red line shows the result of linear regression and the resulting equation is shown.
